# Characterization of mycobacteriophage Adephagia cytotoxic proteins

**DOI:** 10.1093/g3journal/jkae166

**Published:** 2024-07-20

**Authors:** Krista G Freeman, Michael J Lauer, Danny Jiang, Jennifer Roscher, Sterling Sandler, Nicholas Mercado, Robert Fryberger, Julia Kovalski, Abigail R Lutz, Lee E Hughes, Andrew P VanDemark, Graham F Hatfull

**Affiliations:** Department of Biological Sciences, University of Pittsburgh, Pittsburgh, PA 15260, USA; Department of Biological Sciences, University of Pittsburgh, Pittsburgh, PA 15260, USA; Department of Biological Sciences, University of Pittsburgh, Pittsburgh, PA 15260, USA; Department of Biological Sciences, University of Pittsburgh, Pittsburgh, PA 15260, USA; Department of Biological Sciences, University of Pittsburgh, Pittsburgh, PA 15260, USA; Department of Biological Sciences, University of North Texas, Denton, TX 76203, USA; Department of Biological Sciences, University of Pittsburgh, Pittsburgh, PA 15260, USA; Department of Biological Sciences, University of Pittsburgh, Pittsburgh, PA 15260, USA; Department of Biological Sciences, University of Pittsburgh, Pittsburgh, PA 15260, USA; Department of Biological Sciences, University of North Texas, Denton, TX 76203, USA; Department of Biological Sciences, University of Pittsburgh, Pittsburgh, PA 15260, USA; Department of Biological Sciences, University of Pittsburgh, Pittsburgh, PA 15260, USA

**Keywords:** mycobacteriophage, cytotoxic genes, toxin–antitoxin

## Abstract

*Mycobacterium* phage Adephagia is a cluster K phage that infects *Mycobacterium smegmatis* and some strains of *Mycobacterium* pathogens. Adephagia has a siphoviral virion morphology and is temperate. Its genome is 59,646 bp long and codes for one tRNA gene and 94 predicted protein-coding genes; most genes not associated with virion structure and assembly are functionally ill-defined. Here, we determined the Adephagia gene expression patterns in lytic and lysogenic growth and used structural predictions to assign additional gene functions. We characterized 66 nonstructural genes for their toxic phenotypes when expressed in *M. smegmatis*, and we show that 25 of these (38%) are either toxic or strongly inhibit growth, resulting in either reduced viability or small colony sizes. Some of these genes are predicted to be involved in DNA metabolism or regulation, but others are of unknown function. We also characterize the HicAB-like toxin–antitoxin (TA) system encoded by Adephagia (gp91 and gp90, respectively) and show that the gp90 antitoxin is lysogenically expressed, abrogates gp91 toxicity, and is required for normal lytic and lysogenic growth.

## Introduction

Bacteriophage genomes are characterized by densely packed genes, an average gene size that is smaller than their host bacterial genes, and an abundance of genes of unknown function (Brussow and Hendrix 2002; Hendrix 2003; Hatfull and Hendrix 2011). A collection of >2,300 sequenced genomes of phages isolated on a single strain of *Mycobacterium smegmatis* mc^2^155 reveals them to span considerable genetic diversity (Hatfull 2020). All are double-stranded DNA (dsDNA) tailed phages and morphologically are either siphophages or myophages (Hatfull 2020; Hosseiniporgham and Sechi 2022). Based on genomic similarities, they are grouped in clusters (clusters A, B, C, etc.), many of which can be divided into subclusters (subclusters A1, A2, A3, etc.; Hatfull *et al*. 2006, 2010; Gauthier and Hatfull 2023). The current genomes assemble into 34 clusters and there are six “singletons,” each with no close relative (Russell and Hatfull 2017). The overall diversity is heterogenous, with few representatives of some clusters (clusters X and Y each have only two members) and many of others (i.e. 780 and 412 members of clusters A and B, respectively).

Several recent reports suggest that phages of *Mycobacterium* hosts (mycobacteriophages) have therapeutic potential, and favorable microbiological or clinical outcomes have been observed in several treatments of nontuberculous mycobacterium (NTM) infections (Dedrick, Guerrero Bustamante, Garlena, Russell *et al*. 2019a; Dedrick, Smith *et al*. 2023b; Little *et al*. 2022; Nick *et al*. 2022; Hatfull 2023). However, relatively few phages infect any clinical isolate of *Mycobacterium abscessus*, and there is substantial variation in the phage infection profiles of NTM clinical isolates (Dedrick *et al*. 2021; Dedrick, Abad *et al*. 2023a; Gorzynski *et al*. 2023). A similar genomically related subset of phages also infects *Mycobacterium tuberculosis*, although there is much less susceptibility variation among different *M. tuberculosis* strain lineages (Jacobs-Sera *et al*. 2012; Guerrero-Bustamante *et al*. 2021; Opperman *et al*. 2023). In general, the therapeutically useful phages fall within clusters/subclusters A2, A3, G1, K1, K2, K4, and AB, but only subsets of phages within these have the desired host ranges (Dedrick *et al*. 2021; Guerrero-Bustamante *et al*. 2021; Hatfull 2022).

All therapeutic mycobacteriophages have siphophage morphologies and all have defined cohesive genomic termini (Russell and Hatfull 2017; Russell 2018). As such, they share a common genomic architecture in which the virion structure and assembly genes are organized into a long operon typically represented as being transcribed rightward in the genome left arm, as with the prototypical siphophage l of *Escherichia coli* (Sanger *et al*. 1982). Many mycobacteriophages are temperate, usually encoding an integration system located near the genome center, and a repressor that is required for lysogeny and confers superinfection immunity (Hatfull 2020). The right arm genes include some coding for DNA metabolism but most are of unknown function; many are likely not essential for phage lytic growth (Dedrick *et al*. 2013).

**Fig. 1.**
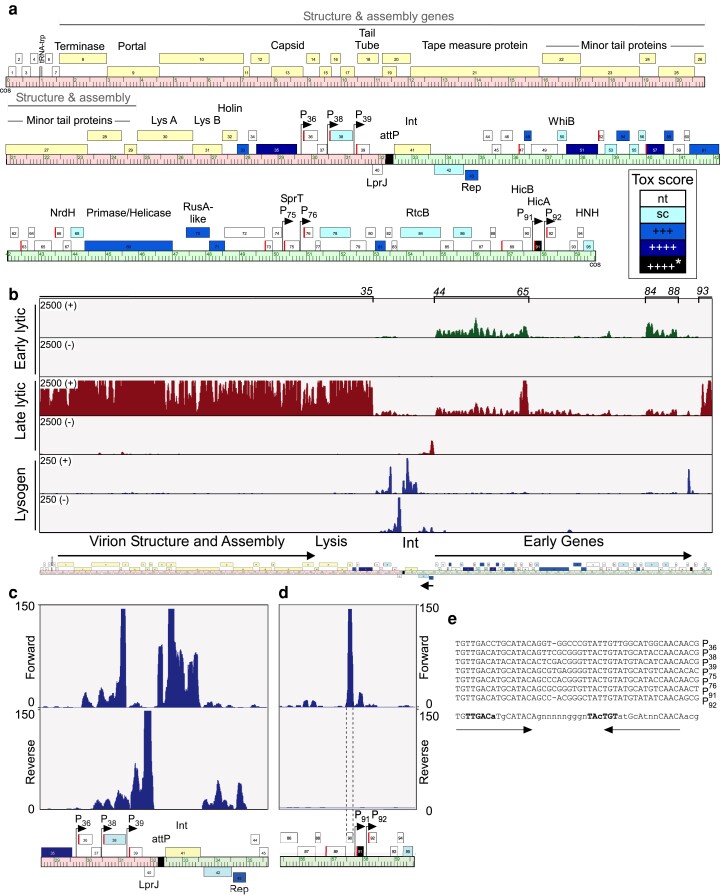
Mycobacteriophage Adephagia genome organization and expression. a) The Adephagia genome is shown as a ruler, with genes shown as boxes above or below the ruler (indicating right- or leftward transcription, respectively). The left arm (from *cos* to *attP*) is shaded light red and the right arm (*attP* to *cos*) is shaded light green. Putative protein functions are indicated above the corresponding genes, where known. The virion structure and assembly genes and the lysis cassette are indicated. The genes are colored in shades of blue according to the cytotoxic effect of the expressed protein, as indicated with the tox score scale (nt, nontoxic; sc, small colony phenotype; +++, reduction in EOP of 10^−3^; ++++, reduction in EOP of 10^−4^; ++++*, reduction in EOP of 10^−4^ in an integrative vector). Genes that were not tested for cytotoxicity are colored light yellow. Genes with a SAS are shown with a red bar at the start of the gene. The positions of 7 ESASs, each containing a putative promoter, are indicated with arrows, and the predicted promoters are indicated by the closest downstream gene. b) Transcription of the Adephagia genome. Sequencing reads obtained from RNA isolated during Adephagia early lytic infection (30 minutes; green) and Adephagia late lytic infection (210 minutes; red), as well as from an *M. smegmatis* mc^2^155(Adephagia) lysogen (blue), are mapped onto the Adephagia genome depicted below. Reads mapping forward (+) and reverse (−) strands are shown as indicated. Note that the read scale maximum is 2,500 for the lytic conditions, but only 250 for the lysogen. c) Expanded view of RNA-seq reads of the lysogenic sample in the central genomic region of the Adephagia genome. d) Expanded view of RNA-seq reads of the lysogenic sample in the gene *86*–*95* region. e) Extended SAS motifs in Adephagia with putative promoter sequences for genes *36*, *38*, *39*, *75*, *76*, *91*, and *92* as indicated to the right. A consensus sequence is shown at the bottom with putative −35 and −10 hexamer sequences shown in bold type; bases conserved in all sequences are shown in upper case and in lower case if present in at least 5 of the genomes.

Cluster K phages, a number of which are therapeutically useful, form a relatively large group (185 members) divided into eight subclusters (K1–K8; Russell and Hatfull 2017). All of the cluster K phages have siphoviral virion morphologies and are temperate, although some are naturally occurring clear plaque derivatives (Pope *et al*. 2011). The genome left arms contain the virion structure and assembly genes, and the right arms contain the early lytic genes (Rybniker *et al*. 2010; Pope *et al*. 2011; Dedrick, Guerrero Bustamante, Garlena, Pinches *et al*. 2019b; also see Fig. 1a). The *attP*-*integrase* (*int*) cassette is located near the middle of the genome, and the immunity repressor is situated nearby and is leftward transcribed (Pope *et al*. 2011; Petrova *et al*. 2015). The cluster K genomes have several short, conserved sequences including 13-bp start-associated sequences (SASs) located upstream of the translation start codons of 13–19 genes, usually within 3–9 bp of the start codon. These are presumably involved in regulating translation initiation of these genes, although their specific role is ill-defined, and the conservation extends beyond the canonical ribosome binding site (Pope *et al*. 2011). A subset of the SAS-associated genes also has a ∼50 bp upstream extended start-associated sequence (ESAS) characterized by a 17-bp imperfectly conserved inverted repeat sequence separated by 12–14 bp (Pope *et al*. 2011). The role of the ESAS sequence is also unclear.

Screening phage genes for toxicity when expressed in the bacterial host is a useful approach to understanding gene function, and 10–20% of nonstructural genes exhibit some degree of cytotoxicity (Liu *et al*. 2004; Ko and Hatfull 2020). Phage gene-mediated toxicity does not likely reflect the gene's role in lytic growth, but at least in some instances reflects inactivation of essential host functions that competing phages need for infection (Ko and Hatfull 2018). Toxicity screens for the genes encoded by mycobacteriophages Waterfoul and Hammy—grouped in subclusters K5 and K6, respectively—showed that 15–16% had strong toxic phenotypes, and similar numbers had milder toxicities (Heller *et al*. 2022; Amaya *et al*. 2023). These phages share about 50% of their genes, but only ∼25% of these showed similar phenotypes (toxicity or lack of toxicity). Phenotypic differences likely result from differences in expression levels, stabilities of expressed proteins, different affinities for target host molecules, or differences in evaluating toxicity, which can range from no growth to reductions in colony size in the presence of inducer. To fully characterize the toxic proteins encoded by mycobacteriophages, it is helpful to test representative phages from different clusters and subclusters.

Here, we characterize the nonstructural genes of *Mycobacterium* phage Adephagia. Adephagia is a previously described mycobacteriophage grouped in cluster K1, has a siphophage virion morphology, is temperate (Pope *et al*. 2011), and is proposed to have therapeutic utility for *Mycobacterium* infections (Guerrero-Bustamante *et al*. 2021). It has a 59.6-kbp genome (accession number JF704105) that codes for 95 predicted protein-coding genes, of which 22 are predicted to be required for virion structure and assembly (Pope *et al*. 2011; see Fig. 1a). It shares only ∼50% of its genes with phages Waterfoul (subcluster K5) and Hammy (subcluster K6; Heller *et al*. 2022; Amaya *et al*. 2023). Of the 66 Adephagia genes tested, 25 (39%) showed growth inhibition when induced, ranging from no growth to small colony size. This represents a distinct but overlapping set of toxic genes from those reported in the related phages. The most toxic gene is *91*, which is part of a HicAB-like toxin–antitoxin (TA) system. We show that gp90 acts as an antitoxin and that gp91 expression in the absence of gp90 prevents lytic phage growth in a temperature-dependent manner.

## Materials and methods

### Bacterial strains

*M. smegmatis* mc^2^155 strains were grown on Middlebrook 7H10 solid medium and in liquid culture in Middlebrook 7H9 supplemented with albumin dextrose complex (ADC) at 37°C (Snapper *et al*. 1990). For protein expression and solubility assays, Rosetta2 *E. coli* was cultured in ZYP-5052 autoinduction media [10-g/L tryptone, 5-g/L yeast extract, 1-mM MgSO_4_, 1× trace metals (Teknova T1001), 200-mM PO_4_, 25-mM SO_4_, 50-mM NH_4_, 100-mM Na, 50-mM K, 0.5% glycerol, 0.05% glucose, 0.2% alpha-lactose] supplemented with selective antibiotics as required. Plasmids were propagated and stored in NEB-5a *E. coli* (New England Biosciences) in Luria–Bertani (LB) media with selective antibiotic markers.

### Plasmid constructions

Details of individual plasmids are reported in Supplementary Table 1 in Supplementary File 1. Plasmid vectors pKF7 and pKF8 (integration-proficient and extrachromosomal, respectively) carry anhydrotetracycline (ATc)-inducible promoters and were derived from pCCK41 and pCCK11, respectively (Ko and Hatfull 2018), by removal of the mCherry gene and insertion of a Hind III restriction site. The plasmids were digested with Hind III, and the PCR-amplified Adephagia genes (or the genes Fruitloop_*52* and mCherry as controls) were inserted by Gibson assembly. Plasmid vector pML5 (an integration-proficient plasmid with an hsp60 promoter) was derived from pLO74 (Oldfield and Hatfull 2014), and Adephagia gene *90* was inserted to create the complementing strain pDJ18. Plasmids for protein expression were derived from a pET28a-based vector carrying a 10× His tag fused to an mRuby2 fluorescent protein gene sequence with a C-terminal linker sequence (Zalewski *et al*. 2016). The vector was linearized with BamHI restriction digestion, and Adephagia genes, amplified by PCR, were inserted with Gibson assembly. For all plasmid constructions, reactions were transformed into *E. coli* NEB 5a chemically competent cells (New England Biosciences), and colony PCR was used to identify colonies carrying candidate plasmids. Transformants were cultured in LB with selective antibiotics, and DNA was isolated and sequenced to confirm the desired construction.

### Construction of Adephagia mutants using BRED

Mutant phages Adephagia Δ*91*, Adephagia Δ*90*Δ*91*, and Adephagia Δ*90* were constructed using bacteriophage recombineering of electroporated DNA (BRED; Marinelli *et al*. 2008). To construct Adephagia Δ*91* and Adephagia Δ*90*, wild-type (WT) Adephagia DNA was PCR amplified to produce 250-bp fragments upstream and downstream of the targeted gene, retaining 21 nucleotides at both the 5′ and 3′ ends of the gene to be deleted. The fragments were joined by Gibson assembly to create a synthetic substrate that lacks most of the targeted gene sequence (all but the first and last 21 nucleotides). To make the substrate for the Adephagia Δ*90*Δ*91* mutant, the upstream 250-bp fragment for the Adephagia Δ*90* reaction was assembled with a downstream fragment amplified using Adephagia Δ*91* genomic DNA as the template. The resulting substrate contains 229 bp of homology upstream of gene *90*, the 5′ and 3′ 21 bp of gene *90*, the intergenic space between genes *90* and *91*, the 5′ and 3′ 21 bp of gene *91*, and 229 bp of downstream homology. Two hundred nanograms of the PCR-amplified substrate and 200 ng of either WT (for Adephagia Δ*91* and Adephagia Δ*90*) or Adephagia Δ*91* (for Adephagia Δ*90*Δ*91*) genomic DNA were transformed into recombination-proficient electrocompetent *M. smegmatis* mc^2^155; for Adephagia Δ*90*, the recombineering cells also contained pDJ18, a plasmid constitutively expressing gene *90*. The transformation mix was recovered at 37°C in 7H9 medium supplemented with ADC and 1-mM CaCl_2_ for an hour and was then plated onto 7H10 agar plates on a lawn of either WT *M. smegmatis* mc^2^155 (for Adephagia Δ*91* and Adephagia Δ*90*Δ*91*) or *M. smegmatis* mc^2^155 carrying the complementing plasmid pDJ18 (for Adephagia Δ*90*). Mixed primary plaques were picked after a 24-hour incubation at 37°C, PCR amplified to determine those with the targeted deletion, and then plated for secondary plaques on lawns of either WT *M. smegmatis* mc^2^155 (for Adephagia Δ*91* and Adephagia Δ*90*Δ*91*) or *M. smegmatis* mc^2^155 carrying the complementing plasmid pDJ18 (for Adephagia Δ*90*). After 24 hours of growth at 37°C, the secondary plaques were picked, PCR verified, Sanger sequenced, and propagated into lysates on appropriate strains. Mutant phages were completely sequenced using previously described methods (Russell 2018) and shown to not have any off-target changes, although the mutants and the parent phage used have a single nucleotide polymorphism at G4958A relative to the previously published sequence.

### Characterization of Adephagia Δ*90* derivatives that plaque on a noncomplementing strain

Five independent lysates of Adephagia Δ*90* were produced by plating secondary plaques from the BRED reaction onto the complementing strain to form lawns with confluent lysis. These were flooded with phage buffer (10-mM Tris, pH 7.5, 10-mM MgSO_4_, 68-mM NaCl), harvested, and filtered. The lysates were each plated on the complementing strain for single plaques, and 2 well-isolated plaques from each lysate were picked into 100 μL of phage buffer; the entire sample was plated onto a noncomplementing strain (*M. smegmatis* mc^2^155) which had been concentrated 10-fold prior to plating and incubated at 37°C for 24 hours. Isolated plaques were picked from these plates and the *90*–*91* region was amplified by PCR; these amplicons were Sanger sequenced. The plaques were also amplified on the complementing strain and Illumina sequenced.

When Sanger-sequencing PCR products of the Adephagia Δ*90* derivative that plaque on a noncomplementing strain, gene *91* mutations were identified in more than half (12/20) of these. Several had WT sequences, but a sequence near the beginning of gene *91* causes a strong stop in the Sanger-sequencing reactions. Illumina sequencing of the complete genomes showed that most (5/7) of these genomes have mutations immediately upstream of gene *91*, presumably interfering with gene *91* expression.

### Site-directed mutagenesis

Primers containing desired mutations were used for site-directed mutagenesis of parental plasmids to create pDJ15 (Adephagia gp91 G28C), pDJ16 (Adephagia gp91 H31A), and pDJ17 (Adephagia gp90 T31V) as follows. Ten nanograms of either pDJ14 (*Adephagia_91*) or pDJ19 (*Adephagia_90)* were amplified with mutagenesis primers and 2× Q5 MasterMix (NEB) for 28 PCR cycles. The amplicon was recircularized with Kinase Ligase DpnI Enzyme Mix (New England Biolabs) and then transformed into *E. coli* NEB 5a chemically competent cells (New England Biosciences). DNA was isolated from transformants and sequenced to confirm the mutations are present.

### RNA-seq

A culture of *M. smegmatis* mc^2^155 was grown to mid-logarithmic phase and cells collected by centrifugation at 5,500 × *g* for 3 minutes at room temperature. The supernatant was removed and transferred to a sterile flask. Adephagia lysate was added to the cell pellet at a multiplicity of infection (MOI) of 3. Following adsorption of phage particles at room temperature for 10 minutes, the saved supernatant was added back to the pellet and the cells were resuspended. The sample was incubated at 37°C with shaking for either 30 or 210 minutes. At each timepoint, 2 mL of Adephagia-infected cells were harvested, mixed with RNAProtect (Qiagen), vortexed, and pelleted by centrifugation at 5000 × *g* for 1 minute. The supernatant was removed and the cell pellets were frozen at −80°C until further processing. Cells were similarly prepared from an Adephagia lysogen. Total nucleic acids were extracted from each sample using the RNEasy kit (Qiagen) and then subjected to TURBO DNase treatment (Ambion). rRNA was removed using QIAseq FastSelect (Qiagen), and a cDNA library was prepared with the NEBNext Ultra II Directional RNA Library Prep Kit for Illumina sequencing (New England Biolabs). Libraries were multiplexed on an Illumina MiSeq, analyzed as described previously (Dedrick *et al*. 2017), and visualized with the Integrated Genomics Viewer (Thorvaldsdottir *et al*. 2013).

### Cytotoxicity plate assay and quantification

Two hundred nanograms of plasmid vector pKF8 and its derivatives (Supplementary Table 1 in Supplementary File 1) were transformed into 100-µL electrocompetent *M. smegmatis* mc^2^155. Fruitloop gp52 was used as a toxic control, mCherry as an expression and nontoxic control, and the empty vector as a background control. The transformed cells were recovered in 7H9/ADC without antibiotics for 3 hours at 37°C and then plated on uninduced and induced (100-ng/mL ATc) solid selective media to assay for cytotoxic effects. Colony growth was compared and the Adephagia genes were assigned one of the following cytotoxicity scores: nontoxic (no change in colony count or size), small colony phenotype, or toxic (3–5 log reduction in number of colonies on the induced plate). Transformants carrying plasmids expressing toxic genes were cultured (in biological triplicate) in 7H9/ADC/Tween 80 (0.05%) with selective antibiotics at 37°C for 72 hours with shaking at 250 rpm. These saturated cultures were used to inoculate fresh media, grown with shaking at 37°C until OD_600_ ∼0.8, 10-fold serial diluted, and 6 µL spotted on solid media with or without ATc induced and incubated at 37°C for 72 hours. For transformants with changes in colony size, colonies on uninduced and induced plates were imaged and quantified for the number and surface area of colony-forming units (CFUs) using Fiji (Schindelin *et al*. 2012). The images were converted to 8-bit and thresholded to make a binary image, and then the edge of the plate and colonies on the edges were removed. A watershed processing step was used to separate too-close colonies, and then the particles were analyzed to count and quantify the area of individual colonies. Violin plots were constructed from the surface areas of each colony on the uninduced and induced plates.

For complementation assays with genes *90* and *91*, 200 nanograms each of the following plasmid pairs (Supplementary Table 1 in Supplementary File 1) were cotransformed into 100-µL electrocompetent *M. smegmatis* mc^2^155: pKF7 and pKF208, pDJ14 and pKF8, pDJ14 and pKF208, pKF7 and pKF114, pKF7 and pKF115, pKF7 and pDJ17, pDJ16 and pDJ14, pDJ16 and pDJ17, pDJ15 and pKF208, and pDJ15 and pDJ17. The transformed cells were recovered as above and individual colonies were cultured into liquid media. Subcultures were grown with shaking at 37°C until reaching OD_600_ ∼0.5 and then normalized to OD_600_ = 0.2, 10-fold serially diluted. These dilutions (10 μL) were spotted on ATc-induced (100 ng/mL) and uninduced plates and incubated at 37°C for 72 hours.

### Efficiency of lysogeny

*M. smegmatis* mc^2^155 was cultured to late-log phase (OD_600_ = 1), 10-fold serially diluted, and 100 µL plated on Middlebrook 7H10 agar plates seeded with at least 1 × 10^8^ plaque-forming units (PFUs) of WT Adephagia or its mutant derivatives, and on unseeded media. The plates were incubated at 37°C for 72 hours and imaged. The number of colonies on each plate was quantified with Fiji (Schindelin *et al*. 2012) as described above, and the efficiency of lysogeny was calculated by dividing the number of colonies on phage-seeded plates by the number on unseeded control plates.

### Lysogen stability experiments

Lysogenic strains from the efficiency of lysogeny assay were streak purified (in biological triplicate) onto Middlebrook 7H10 agar plates, then inoculated into liquid media 7H9/ADC/CB/CHX/Tween 80 (0.05%) and grown at 37°C for 72 hours with shaking. These starter cultures (generation 0) were normalized to OD_600_ = 1, and then samples were removed and centrifuged at 11,000*×g* for 2 minutes. One hundred microliters of each supernatant was mixed with 300 μL of *M. smegmatis* mc^2^ 155 and 3 mL of 0.35% MBTA (Middlebrook Top Agar) and spread on solid media and then incubated at 37°C for 24 hours to check for levels of spontaneous phage release. Additionally, the OD-normalized cultures were 10-fold serially diluted, and then 100 µL of the 10^−5^ dilutions were spread onto Middlebrook 7H10 agar plates seeded with 1 × 10^8^ PFUs of Adephagia Δ*43* or onto unseeded media. The number of colonies on each plate was quantified with Fiji (Schindelin *et al*. 2012), and the percentages of colonies containing the prophage (*N*_seeded_/*N*_unseeded_) were calculated and plotted for each lysogenic strain. These assays were repeated 3 times with sequential subcultures (each diluted 1:10,000) and regrowth to saturation (13 generations elapsed for each subculture).

### Protein structure modeling and bioinformatic predictions

Protein structural models were predicted with AlphaFold2 (Jumper *et al*. 2021) and then queried in the structural alignment databases DALI (Holm 2022) and Foldseek (van Kempen *et al*. 2024). For DALI, functional hits were considered with *z*-scores of ≥5.0. For Foldseek, functional hits were considered with an *e*-value of <1 and a probability of >70%. The Adephagia gp90/gp91 complex was predicted with AlphaFold-Multimer (Evans *et al*. 2022).

### Protein solubility experiments

Adephagia proteins were expressed Rosetta2 *E. coli* cells by culturing a colony in 1 mL of autoinduction media (detailed in the “Bacterial strains” section) at 17 or 37°C with 300 rpm shaking until saturation. Cells were pelleted at 7,000 rpm for 2 minutes and then frozen at −80°C. Pellets were removed from frozen storage and thawed at room temperature for 5 minutes before being subjected to two freeze–thaw cycles by freezing for 15 minutes in a dry ice/ethanol bath followed by incubation at 37°C for 1 minute. Pellets were then resuspended in 300-μl lysis buffer (20-mM Tris at pH = 8, 500-mM NaCl, 5% glycerol, 2-mM BME, 1-mg/mL lysozyme, 1 μL/mL each of pepstatin, aptrotin, and leupeptin) and incubated at room temperature for 20 minutes and then at 30°C for 10 minutes. DNase I and MgSO_4_ were added to 0.1 mg/mL and 30 mM, respectively, before an additional incubation for 20 minutes at room temperature and 10 minutes at 30°C. From these lysed samples, 100 μL was removed as the whole cell lysate and transferred to a 96-well plate. The remaining samples were centrifuged at 13,000 rpm for 15 minutes at 4°C, and 100 μL of the supernatants were removed and transferred to a 96-well plate. The relative levels of Ruby2 fluorescent protein in the whole cell lysate and supernatant samples were quantified using a plate reader, and the ratios of these reads were calculated. The same whole cell lysate and supernatant samples were mixed with 4× SDS loading buffer (0.2-M Tris, 0.4-M dithiothreitol, 277-mM sodium dodecyl sulfate, 6-mM bromophenol blue, 4.3-M glycerol), boiled for 15 minutes, and loaded onto SDS-PAGE gels to visualize the expressed protein populations.

### Lysogen superinfection defense experiments

Top-agar lawns of *M. smegmatis* mc^2^ 155, lysogenic strains, and *M. smegmatis* mc^2^ 155 carrying a plasmid with an active CRISPR system targeting gene *90* were prepared by mixing 300 μL of bacteria with 3 mL of 0.35% MBTA and spreading on Middlebrook 7H10 agar plates. Ten-fold dilutions of various phages were prepared and spotted onto these prepared lawns, and then the plates were incubated at 37°C for 24 hours.

## Statistical analysis

All statistical analyses were performed with Prism 10. Statistical significance was assessed with unpaired *t*-tests and resulting one-tailed *P*-values are shown in figures for sample pairs that are significantly different.

## Results

### Genome organization and gene expression in mycobacteriophage Adephagia

The Adephagia genome is organized into left (*cos-attP*) and right (*attP-cos*) arms with the *attP-int* cassette close to the center of the genome (Fig. 1a). Gene functions were previously assigned (Pope *et al*. 2011) using homology searches with BLAST and HHpred (Pope and Jacobs-Sera 2018). Most genes are transcribed rightward, with the exception of 3: the repressor (gene *43*); the adjacent downstream gene *42*, which is of unknown function but is strongly predicted to be a membrane protein with 7 predicted transmembrane domains; and gene *40* (Fig. 1a). Gene *40* codes for a predicted LprJ-like lipoprotein with an N-terminal signal sequence. There is a single tRNA gene (gene *5*) located near the extreme left end of the genome (Fig. 1a).

Transcriptomic analysis confirms that early lytic expression begins upstream of gene *44*, such that divergent transcription from within the *43*–*44* intergenic region expresses the repressor and the early lytic genes (Fig. 1b). SigA-like promoters cannot be readily identified bioinformatically in this region, and we note that only low levels of repressor transcription are observed in a lysogen (Fig. 1, b and c). The early lytic region appears to extend from gene *44* through to gene *88*, but the transcript levels are quite heterogenous, with the gene *66*–*83* region at lower or near undetectable levels and the *44*–*65* and *84*–*88* regions at higher levels (Fig. 1b). Late transcription appears to initiate between genes *92* and *93* and extends through *cos* into the left arm, ending after gene *35* (Fig. 1b); we note that this includes the virion structure and assembly genes (*6*–*29*) as well as the lysis cassette (*30*–*32*; Fig. 1, a and b).

In a lysogen, in addition to expression of the repressor (gene *43*) and the adjacent membrane protein gene (*42*)—albeit at low levels—we also see relatively strong expression of integrase (gene *41*) and genes *36–38*, all rightward transcribed (Fig. 1c). On the reverse strand, gene *40* is relatively strongly expressed, and the leftward expression appears to extend at lower levels through to the position of gene *37*, antisense to genes *37*–*39*. At the right end of the genome, there is relatively strong lysogenic expression of gene *90*, which is predicted to be a HicB-like antitoxin (Fig. 1, a and d). We note that there are only low expression levels of gene *91*, which codes for a putative HicA-like toxin (Fig. 1, a and d; Table 1).

**Table 1.** Adephagia genes tested for toxicity.

Gene	Tested?*^a^*	Function*^b^*	Toxic*^c^*	DALI *z*-score*^d^*	Foldseek *e*-value*^e^*
1	Yes	Hypothetical	No	No informative match; N/A	Translocase (SecD); 3.93e-1
2	Yes	Hypothetical	No	No informative match; N/A	No informative match; N/A
3	Yes	Hypothetical	No	No informative match; N/A	Terminase, large subunit; 5.04e-3
4	Yes	Hypothetical	No	No informative match; N/A	No informative match; N/A
6–29	No	Virion	N/A	N/A	N/A
30	No	Lysin A	N/A	N/A	N/A
31	No	Lysin B	N/A	N/A	N/A
32	No	Holin	N/A	N/A	N/A
33	Yes	Membrane	+++	No informative match; N/A	No informative match; N/A
34	Yes	Hypothetical	No	No informative match; N/A	HTH-IS21 transposase domain; 3.25e-1
35	Yes	Exonuclease	++++	Sliding clamp; 18.4	DNA pol domain; 6.09e-9
36	Yes	Hypothetical	No	No informative match; N/A	DNA binding domain, Xis family; 1.97e-4
37	Yes	Hypothetical	No	No informative match; N/A	No informative matches; N/A
38	Yes	Hypothetical	sc (0.57)	No informative match; N/A	No informative matches; N/A
39	Yes	Hypothetical	No	HfQ; 8.8	HfQ; 4.72e-2
40	Yes	Hypothetical	No	No informative match; N/A	Lipoprotein LprJ; 1.81e-3
41	No	Integrase	N/A	N/A	N/A
42	Yes	Membrane	sc (0.14)	No informative match; N/A	Membrane; 5.66e-7
43	Yes	Repressor	+++	PrgX; 5.7	Cro/C1-type HTH domain; 8.93e-4
44	Yes	Cro	No	Transcription regulator; 7.4	Cro/C1-type HTH domain; 6.16e-3
45	Yes	Xis?	No	MerR family regulator; 7.4	Excisionase; 7.40e-4
46	Yes	Hypothetical	No	No informative match; N/A	No informative match; N/A
47	Yes	Membrane	No	No informative match; N/A	No informative match; N/A
48	Yes	Hypothetical	+++	No informative match; N/A	No informative match; N/A
49	Yes	MerR-like	No	MerR family regulator; 7.7	HTH_17 domain; 4.35e-1
50	Yes	WhiB	sc (0.05)	WhiB1; 9.5	WhiB1; 2.45e-4
51	Yes	DUF3310	++++	No informative match; N/A	DUF3310; 2.1e-3
52	Yes	Membrane	No	No informative match; N/A	Lipoprotein; 2.7e-2
53	Yes	Membrane	sc (0.03)	SubA; 7.4	Pertussis toxin subunit; 5.08e-2
54	Yes	Hypothetical	+++	Gam-like; 6.5	PHB domain; 4.61e-1
55	Yes	Hypothetical	sc (0.13)	No informative match; N/A	Endonuclease; 8.99e-5
56	Yes	Hypothetical	sc (0.12)	No informative match	No informative match; N/A
57	Yes	DnaQ	++++	Ribonuclease T; 17.5	3′–5′ exonuclease; 6.15e-21
58	Yes	Hypothetical	+++	No informative match; N/A	No informative match; N/A
59	Yes	Hypothetical	No	No informative match; N/A	DUF1523; 7.66e-1
60	Yes	Hypothetical	No	No informative match; N/A	No informative match; N/A
61	Yes	Cas4-like	+++	Nuclease; 11.8	Cas4-like; 3.68e-3
62	Yes	Hypothetical	No	No informative match; N/A	Protease; 2.40e-1
63	Yes	Hypothetical	No	No informative match; N/A	Ring-type domain; 9.42e-3
64	Yes	Hypothetical	No	No informative match; N/A	No informative match; N/A
65	Yes	Hypothetical	No	RuvB-like; 5.5	Phage Pfi helix stabilizer; 3.14e-2
66	Yes	NrdH	No	NrdH; 13.5	NrdH; 9.61e-7
67	Yes	Hypothetical	No	No informative match; N/A	No informative match; N/A
68	Yes	Hypothetical	sc (0.58)	HNH; 6.1	HNH; 1.03e-1
69	Yes	Primase/Helicase	+++	Primase; 19.5	Primase; 2.09e-19
70	Yes	RusA	+++	RusA; 11.3	RusA; 1.77e-15
71	Yes	Hypothetical	+++	No informative match; N/A	HTH Cro/C1-type domain; 2.31e-2
72	Yes	Hypothetical	No	Terminase small subunit; 7.4	PhnA; 1.88e-10
73	Yes	Hypothetical	No	No informative match; N/A	No informative match; N/A
74	Yes	Hypothetical	No	No informative match; N/A	DUF2158; 1.47e-1
75	Yes	SprT	No	SprT; 11.2	SprT; 1.90e-23
76	Yes	Membrane	No	CHAD domain protein; 9.9	Pep-cys S-nitrosylase GAPDH; 9e-1
77	Yes	Hypothetical	No	No informative match; N/A	Nudix hydrolase; 4.31e-1
78	Yes	Membrane	sc (0.01)	No informative match; N/A	HTH domain; 3.30e-10
79	Yes	Hypothetical	No	No informative match; N/A	No informative match; N/A
80	Yes	Hypothetical	No	DNA binding protein; 6.9	DNA binding protein; 8.36e-3
81	Yes	Hypothetical	+++	No informative match; N/A	50S ribosomal protein L24; 1.79e-1
82	Yes	Hypothetical	No	No informative match; N/A	RNA polymerase subunit; 3.92e0
83	Yes	Hypothetical	No	No informative match; N/A	No informative match; N/A
84	Yes	RtcB	sc (0.17)	RtcB; 43.1	RtcB; 9.23e-69
85	Yes	Hypothetical	No	Terminase-like domain; 9.2	Terminase-like domain; 8.37e-3
86	Yes	Hypothetical	sc (0.05)	No informative match	No informative match; N/A
87	Yes	Hypothetical	No	No informative match	No informative match; N/A
88	Yes	Hypothetical	No	Kinase; 5.4	No informative match; N/A
89	Yes	Hypothetical	No	Glycosyl transferase domain	Accessory sec system, Asp1; 7.12e-2
90	Yes	HicB-like	No	HicB; 5.0	HicB; 1.03e-6
91	Yes	HicA-like	++++*	HicA; 5.1	mRNA interferase HicA; 4.57e-4
92	Yes	Hypothetical	No	No informative match; N/A	No informative match; N/A
93	Yes	Hypothetical	No	No informative match; N/A	No informative match; N/A
94	Yes	Hypothetical	No	No informative match; N/A	No informative match; N/A
95	Yes	HNH	sc (0.09)	HNH; 6.6	HNH; 8.48e-12

^*a*^Predicted function, where known.

^*b*^Tested by cloning and determining growth impact.

^*c*^Toxicity to *M. smegmatis* is reported with “+++,” “++++” indicating 3 or 4 log reduction in viability, respectively, or “++++*” indicating such strong cytotoxicity that transformants could only be recovered on an integrated vector; sc: small colony phenotype with the size ratio of colonies on induced vs uninduced plates shown in parenthesis.

^*d*^The structure of each gene product was predicted using AlphaFold and then compared to the PDB database using DALI. The best scoring informative match is shown. No informative match is shown if only lower scoring *z*-scores were reported, or if several functionally distinct proteins were reported with similar *z*-scores. The *z*-score for the indicated match is shown.

^*e*^The predicted structure compared to several AlphaFold databases using Foldseek; hits are shown after search against several databases (AFDB-Proteome, AFDB-SwissProt, AFDB50, or PDB100), and *e*-value is reported after functional hit. No informative match is shown if the probability is >70% (not reported) and *e*-value is <1.0.

Mycobacteriophage transcriptional promoters are poorly defined, although some have SigA-like recognition sequences, with −10 and −35 hexamers similar to those of *E. coli* s-70 promoters (Nesbit *et al*. 1995; Brown *et al*. 1997). Motif searches of the Adephagia genome identified 7 instances of a conserved 46–47-bp sequence containing putative SigA-like promoter, all located intergenically in the Adephagia genome (Fig. 1, a and e). These correspond to the ESAS reported previously (Pope *et al*. 2011), and in addition to SigA-like −10 and −35 hexamers spaced 17–18 bp apart, the motif contains an imperfectly conserved 15-bp inverted repeat (Fig. 1e). Some of the genes located immediately downstream of these motifs (e.g. *36*–*38*, *91*) have detectable (albeit low, for some) levels of lysogenic expression, but others (e.g. 75, 76, and 92) are not lysogenically expressed. It is plausible that expression is repressed by a transcriptional regulator binding to the inverted repeats. The identity of such a putative regulator is unclear, although it is unlikely to be the superinfection immunity repressor gp43, as the motif is absent from the gene *43*–*44* intergenic region where early lytic transcription initiates.

### Overexpression of Adephagia predicted proteins

We amplified, cloned, and expressed a subset of Adephagia genes in *E. coli* to explore the possibility of future experimental structural characterization. A subset of 49 genes were cloned into a pET28-based vector to express fusion proteins with mRuby2 fused to the N-termini of Adephagia proteins. *E. coli* transformants were cultured in autoinduction media at either 17 or 37°C until the cultures were deeply pink in color. The cells were harvested, lysed, and then split for 1 whole cell lysate sample and 1 soluble fraction which was clarified by centrifugation. The mRuby2 fluorescent signal was measured in both samples to determine the percentage of soluble fusion protein in each whole cell lysate (Supplementary Fig. 1a in Supplementary File 1). When expressed at 37°C, 19 of the 49 lysates had at least 30% soluble protein, whereas expression at 17°C yielded 31 proteins with at least 30% soluble protein (Supplementary Fig. 1a in Supplementary File 1). For proteins expressed at 17°C, the proteins were also separated by SDS-PAGE to verify that the pink hue in the soluble fraction is from the fusion protein and not cleaved mRuby alone (Supplementary Fig. 1a in Supplementary File 1). These data suggest that many of these proteins can be targeted for crystallographic solutions, although the availability of AlphaFold provides an alternative approach for predicting structures and potentially functions.

### Predictions of additional Adephagia gene functions

Previous bioinformatic analyses identified putative functions for the virion structure and assembly genes (*6*–*29*), the lysis cassette (genes *30*–*32*), the repressor/immunity system (genes *41*, *43*, and *45*), and 9 other genes (*50*, *57*, *66*, *69*, *70*, *75*, *78*, *84*, and *95*; Table 1, Fig. 1a; Pope *et al*. 2011). In an effort to identify additional functions, we used AlphaFold2 (Jumper *et al*. 2021) to predict the structures of the functionally ill-defined Adephagia genes (Supplementary Fig. 2 in Supplementary File 1) and then used DALI (Holm 2022) and Foldseek (van Kempen *et al*. 2024) to identify structural homologues (Table 1). Relatively few new functions could be confidently predicted, but gp39 is Hfq-like, gp55 is a putative endonuclease, gp71 has a predicted DNA binding motif, and gp78 is a membrane protein that also has a C-terminal DNA binding motif. Several other proteins (gp1, gp3, gp34, gp53, gp54, gp62, gp63, gp65, gp68, gp72, gp76, gp77, gp81, gp82, gp85, and gp89) had weaker matches that warrant further investigation but do not provide confident functional assignments (Table 1).

### Identification of Adephagia genes toxic to *M. smegmatis* mc^2^ 155 growth

**Fig. 2.**
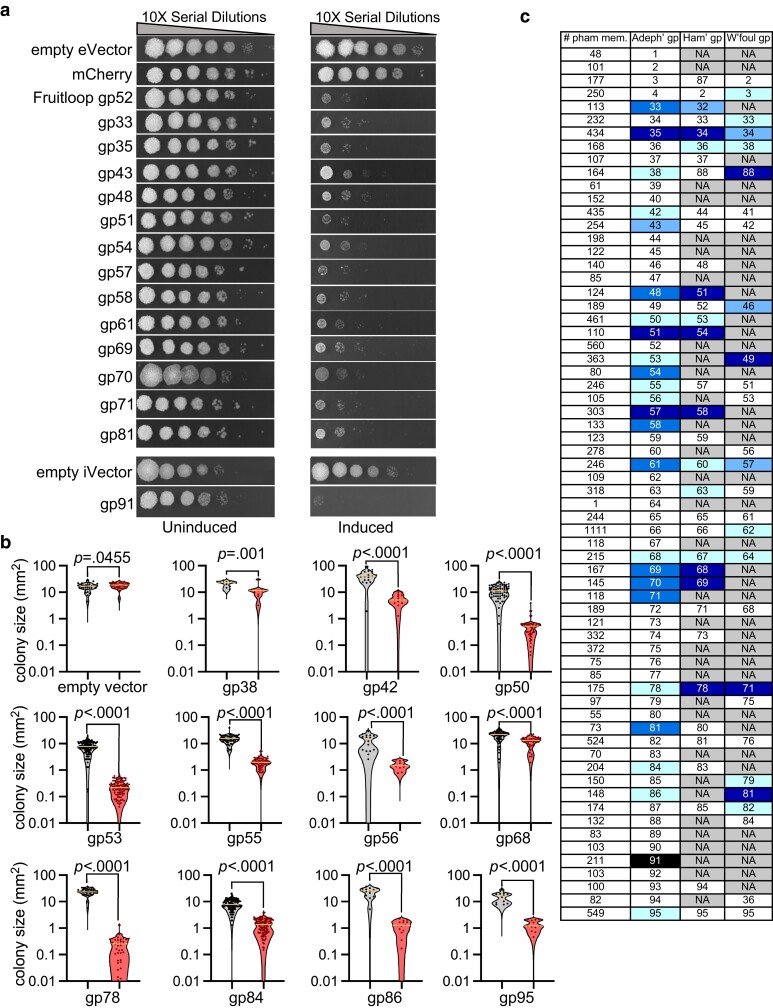
Cytotoxic protein assay. a) Toxicity assays are shown for a series of Adephagia genes expressed from an ATc-inducible promoter. Cultures of *M. smegmatis* strains expressing Adephagia proteins as indicated at the left were 10-fold serially diluted and plated on solid media without (uninduced) or with (induced) ATc (100 ng/mL). Strains expressing either mCherry or Fruitloop gp52 are shown as controls. Adephagia gp91 was expressed from integration-proficient vector pKF7; all others were expressed from extrachromosomal vector pKF8. Empty vectors of pKF8 (empty eVector) and pKF7 (empty iVector) are shown as controls. Solid media plates were incubated at 37°C for 3 days. b) Violin plots showing the distributions of colony sizes for strains expressing Adephagia genes as indicated below each plot. *M. smegmatis* cultures were diluted and plated on solid media with or without ATc inducer for single colonies and imaged after 5 days of incubation at 37°C. Statistical significance was assessed by an unpaired *t*-test of the colony size distributions on uninduced (gray) and induced (red) media; *P*-values are indicated. c) A summary of gene toxicities in phages Adephagia, Hammy, and Waterfoul. Each tested Adephagia gene, indicated in the second column, encodes a protein that belongs to a phamily of homologues; the current (as of February 2024) number of phamily members is indicated in the first column. Phamily construction is described in detail elsewhere (Gauthier *et al*. 2022). The homologous gene numbers for Hammy/Waterfoul are shown, or the box is labeled N/A and colored gray if there is no homologue. Cytotoxic and inhibitory gene products are highlighted in shades of blue (as in Fig. 1a) for each phage. A white background indicates that the protein expression had no negative impact on cell growth.

To identify Adephagia genes that are toxic or inhibitory to *M. smegmatis* growth, we selected 66 genes for further characterization, omitting the virion structure and assembly genes (*6*–*29*), the lysis genes (*30*–*32*), and integrase (*41*; Table 1). Each gene was PCR amplified from Adephagia and cloned into an extrachromosomal shuttle plasmid (eVector, pKF8) downstream of a tet-inducible promoter and the ribosome binding site of the phage TM4 capsid gene. *M. smegmatis* transformants were grown in the presence and absence of the ATc inducer on solid media, and the phenotypes were evaluated, using the known toxic gene Fruitloop gp52 (Ko and Hatfull 2018) as a positive control (Table 1, Fig. 2). For 13 genes (*33*, *35*, *43*, *48*, *51*, *54*, *57*, *58*, *61*, *69*, *70*, *71*, and *81*), we observed strong reductions in the efficiencies of plating by at least 3 orders of magnitude (Fig. 2a, Supplementary Fig. 3 in Supplementary File 1). One gene, *91*, was cloned into the eVector and recovered in *E. coli*, but the plasmid (pKF209) did not transform *M. smegmatis* even in the absence of an inducer. We were able to construct an analogous plasmid (pDJ14) using an integration-proficient vector (iVector, pKF7), which does transform *M. smegmatis*; this showed a strong reduction in plating efficiency when induced (Fig. 2a, Supplementary Fig. 3 in Supplementary File 1). For an additional 11 genes (*38*, *42*, *50*, *53*, *55*, *56*, *68*, *78*, *84*, *86*, and *95*), we did not see an evident reduction in the efficiency of plating (EOP), but we observed significant reductions in colony sizes when expression was induced (Fig. 2b, Supplementary Fig. 4 in Supplementary File 1). Quantification of colony sizes shows that growth inhibition can be quite pronounced, with genes *53* and *78* representing the most extreme examples (Fig. 2b, Supplementary Fig. 4 in Supplementary File 1).

Of the 66 genes tested, 25 (38%) were found to be toxic or inhibitory to *M. smegmatis* growth. These include several genes implicated in DNA/RNA metabolism or regulation, including genes *35* (sliding clamp), *43* (repressor), *49* (*merR*-like), *50* (*whiB*), *57* (*dnaQ*), *61* (*cas4*-family nuclease), *69* (primase/helicase), *70* (*rusA*), *84* (*rtcB*), and *95* (HNH motif). Several others code for predicted membrane proteins, including genes *33*, *42*, and *78* (coding for proteins with 1, 7, and 4 predicted transmembrane domains, respectively); however, not all of the predicted membrane proteins are toxic or inhibitory, and gp47, which contains a putative transmembrane domain, and gp52 and gp67, which have putative signal sequences, did not inhibit growth. The gene with the greatest toxicity and only testable in an integration-proficient vector is *91*, coding for a HicA-like toxin.

We compared the Adephagia gene toxicity profiles with those reported previously for phages Hammy and Waterfoul (Heller *et al*. 2022; Amaya *et al*. 2023), although we note that the plasmid system used in those studies differs from that used here. These phages share 50–60% of their genes in pairwise comparisons (Fig. 2c), and there are many examples where the toxicities of homologues in all three phages are in agreement, but several notable departures (Fig. 2c). Of the 66 Adephagia genes tested, 42 have homologues in either Hammy or Waterfoul, and of these 18 were nontoxic in both Adephagia and 1 or both of the other phages (Fig. 2c). However, 8 of the shared Adephagia genes that are nontoxic were reported to have toxic homologues in either Hammy or Waterfoul (or both). There are also examples such as the toxic/inhibitory Adephagia genes *42*, *43*, *55*, *56*, *71*, *81*, *84*, and *95* whose Hammy/Waterfoul homologues are nontoxic. We note that Adephagia gp81, which is quite strongly toxic, shares 74% amino acid identity with nontoxic Hammy gp80. Additionally, although Adephagia gene *38* is growth inhibitory, its Waterfoul homologue (58% amino acid identity) is strongly toxic and its Hammy homologue (81% amino acid identity) is nontoxic. It is clear that related genes in different genomes can exhibit broad ranges of toxic or inhibitory effects.

### Adephagia encodes a HicA-/B-like TA system

**Fig. 3.**
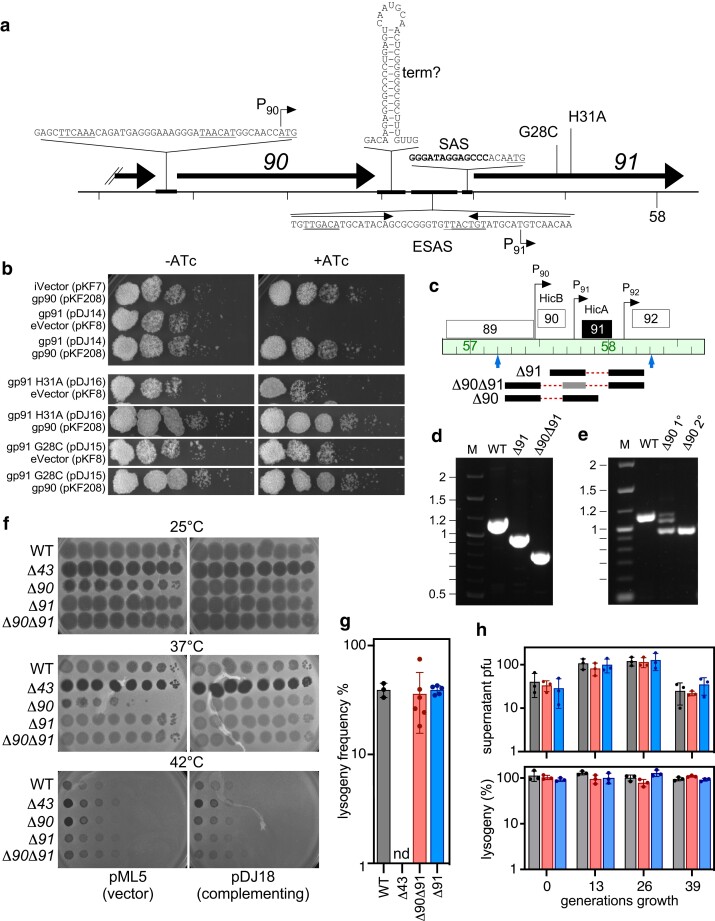
Adephagia gp91–gp90 is a HicAB-like TA system. a) Organization of the gene *90*–*91* region of Adephagia. The Adephagia genome is represented as the thin black line with markers every 100 bp, and the 58 kbp coordinate is indicated. Genes *90*, *91*, and the 3′ end of *89* are shown as black arrows. Shown above and below are key sequence features: A putative promoter for gene *90* (P_90_) is shown that predicts transcription initiation at coordinate 57,504 and use of a leaderless transcript for gene *90*; a putative RNA secondary structure that likely acts as a transcriptional terminator (“term?”); the ESAS sequence with the P_91_ promoter with −35 and −10 sequences underlined and inverted repeats illustrated by arrows; the SAS sequence upstream of gene *91* with the conserved position shown in bold type; the positions of gp91 substitutions G28C and H31A. b) Ten-fold serial dilutions of *M. smegmatis* cultures (OD_600_ = 0.2) carrying plasmids as shown on the left were plated on solid media either with or without ATc inducer and incubated for 3 days. Plasmids are as follows: integration-proficient vector (iVector) and extrachromosomal vector (eVector) are pKF7 and pKF8, respectively. Plasmids pDJ14, pDJ16, and pDJ15 express Adephagia gp91, gp91 H31A, and gp91 G28C, respectively, in the iVector. Plasmid pKF208 expresses gp90 from the eVector. c) A section of the Adephagia genome map as in Fig. 1a, zoomed into the region of genes *90* and *91*. Below the genome ruler are indicated primer locations (blue arrows) and BRED substrates for the creation of deletion mutants (black solid lines indicate regions of homology and red dotted lines indicate deleted portions of the genome). The gray bar in the Δ*90*Δ*91* mutant indicates a region that is retained (see Materials and Methods) d) A gel showing PCR products for WT Adephagia, Adephagia Δ*91*, and AdephagiaΔ*90*Δ*91* amplified with the primers shown in panel c. e) A gel showing PCR products for mixed primary (1°) and pure secondary (2°) plaque picks from the Adephagia Δ*90* BRED reaction amplified with the primers shown in panel c. f) Ten-fold dilutions of lysates of WT Adephagia and the Δ*43*, Δ*90*, Δ*91*, and Δ*90*Δ*91* mutants were spotted onto lawns of *M. smegmatis* mc^2^ 155 carrying plasmids pML5 (empty vector) or pDJ18 (constitutively expressing gp90) as indicated at the bottom and incubated at 3 temperatures as indicated above each set of plates. g) The frequency of lysogen formation on plates seeded with WT Adephagia (gray) and the Δ*43* (no lysogens detected, nd), Δ*90*Δ*91* (red), and Δ*91* (blue) mutants. h) Levels of spontaneous phage release (PFU in 100 μL of supernatant from saturated cultures normalized to OD_600_ = 1), top, and % of CFU in culture belonging to lysogens, bottom, measured every 13 generations (via repeated subculturing) for WT Adephagia (gray) and the Δ*90*Δ*91* (red) and Δ*91* (blue) mutants.

The most toxic of the Adephagia proteins, gp91, is a HicA-like toxin, and a HicB-like antitoxin is encoded by the upstream gene *90*. This antitoxin–toxin organization is typical of class 2 HicAB systems as classified by Gerdes (Fig. 3a; Gerdes 2024). The region contains several features of interest including putative promoters for gene *90* (P_90_) and *91* (P_91_). Gene *91* contains an SAS sequence immediately upstream of the AUG translation start site, although it is one of the 14 identical sequences associated with translation start sites in Adephagia (Pope *et al*. 2011); the role of these is not known (Pope *et al*. 2011). Upstream of the SAS is an ESAS sequence (Fig. 1e) that includes the putative P_91_ promoter, but also the 17-base imperfectly conserved inverted repeat (Fig. 1e). Gene *90* is relatively well-expressed in lysogenic cells (Fig. 1d), presumably from the P_90_ promoter, but transcription ends in the gene *90*–*91* intergenic region (Fig. 1d and 3a). A region predicted to form a 17-base stem with a 7-base loop in the RNA transcript is a candidate for mediating transcription termination or RNA processing (Fig. 3a).

**Fig. 4.**
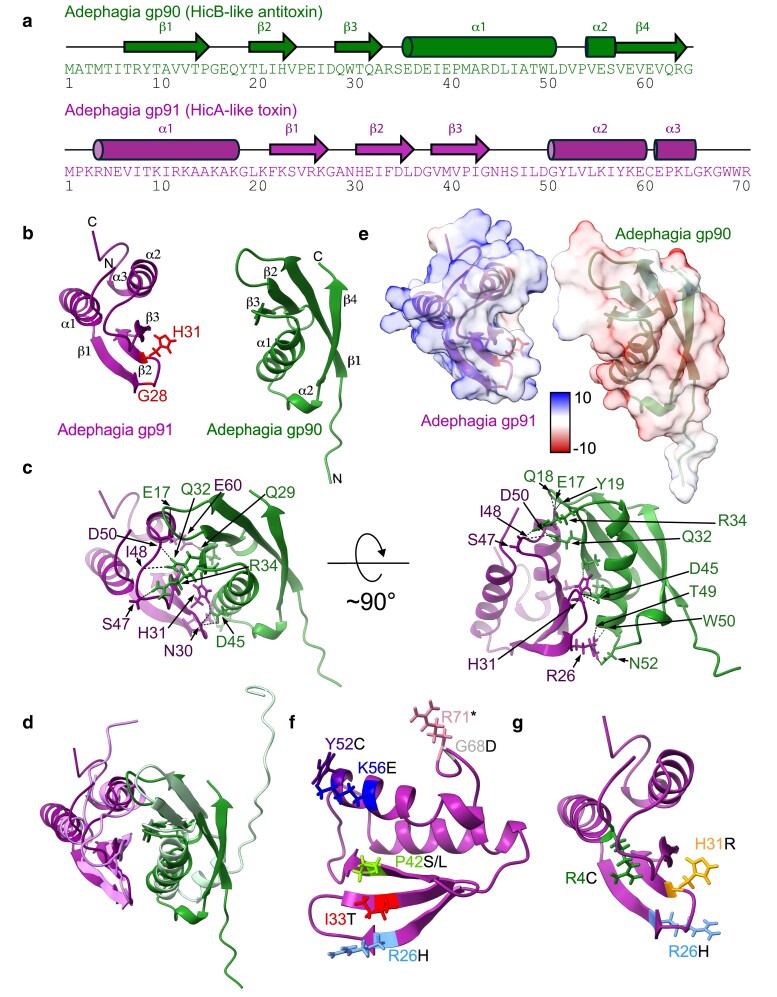
Adephagia gp90 and gp91 structural models. a) Sequences of Adephagia gp90 (green) and gp91 (magenta) with arrows and cylinders shown above the sequence to indicate beta strands and alpha helices, respectively. Numbers below the sequences indicate every 10th residue. b) AlphaFold2-predicted models of Adephagia gp90 (magenta, left) and gp91 (green, right), with features named as in panel a. H31 and G28 are shown with sticks and colored red in the gp91 structure. c) The same models in the same orientation shown in panel b but with semitransparent surfaces showing coulombic electrostatic potential. The color key is shown, with values ranging from +10 to −10 kcal/(mol·*e*). d) A complex of gp90 and gp91 folded with AlphaFold-Multimer, with hydrogen bonds shown with black dotted lines and side chains involved in these bonds shown with sticks. The same gp90/gp91 complex model is shown on the right but rotated 90° into the page. e) The gp90/gp91 complex aligned with AlphaFold2-predicted models of the HicB/HicA proteins encoded by *Campylobacter* sp. RM12654, shown with lighter hues of the same colors. f) Single amino acid substitutions in gp91 are mapped onto the predicted gp91 model; these mutations were isolated from Adephagia Δ*90* plaques grown on a noncomplementing strain of *M. smegmatis* at 37°C. Amino acid side chains of the WT residues are shown with color-coded identifiers; the black letter at the end of each label indicates the substitution. g) Single amino acid substitutions in gp91 were identified in putative Adephagia Δ*90* lysogens, and these are mapped onto the predicted model. Amino acid side chains of the WT residues are shown with color-coded residue identifiers; the black letter at the end of each label indicates the substitution.

To confirm that gp90 acts as an antitoxin, we constructed recombinant strains with either constitutive expression of gp90, inducible expression of gp91, or both (Fig. 3b). Expression of gp91 alone is severely inhibitory to growth as described above, but coexpression of gp90 completely abrogates this toxicity (Fig. 3b). HicA-like proteins typically act to degrade RNA targets, with a catalytically important histidine in the active site and an upstream glycine residue also playing an important role (Makarova *et al*. 2006; Butt *et al*. 2014); protein alignments indicate that H31 and G28 in Adephagia gp91 corresponds to these residues (Fig. 3a, Fig. 4). To determine the role of these residues in Adephagia toxin activity, we constructed mutant plasmids expressing gp91 G28C or H31A substitutions and tested these for toxicity (Fig. 3b). Toxicity is strongly abrogated in both mutants regardless of the presence of the gp90 antitoxin (Fig. 3b).

Although TA systems are not uncommon in bacteriophage genomes, their roles are poorly defined. We note that only low levels of toxin expression are seen in Adephagia lysogenic cells (Fig. 1d), but because gp91 is highly toxic, even low levels of expression could be deleterious in the absence of gp90 expression. As such, the Adephagia TA system could play roles in prophage maintenance by viability loss of spontaneously cured cells; alternatively it could confer defense against heterotypic phage infection as shown for other TA systems (LeRoux and Laub 2022). To explore this latter role, we tested a diverse panel of approximately 60 phages for differences in infection between lysogenic and nonlysogenic strains (Supplementary Fig. 5 in Supplementary File 1). No differences were observed, although heterotypic defense could be phage specific as reported previously (Dedrick *et al*. 2017; Ko and Hatfull 2018; Gentile *et al*. 2019; Montgomery *et al*. 2019), with the targeted phages yet to be identified.

To further characterize the Adephagia TA system, we constructed mutant phage derivatives in which genes *90*, *91*, or both *90* and *91* are deleted from the genome. Deletions of gene *91* (Δ*91*) and the *90*–*91* gene pair (Δ*90*Δ*91*) were readily constructed using BRED engineering (Marinelli *et al*. 2008), and no differences in plaque sizes or morphologies of the mutants were observed (Fig. 3, c, d, and f). Deletion of gene *90* alone was more challenging, and attempts to remove gene *90* using standard approaches were repeatedly unsuccessful, with recovery of mixed plaques containing both WT and mutant alleles but were unsuccessful in purifying the Δ*90* mutant. However, we succeeded in constructing the Δ*90* mutant by using recombinant *M. smegmatis* strains constitutively expressing Adephagia gene *90* to both construct and recover the mutant phage (Fig. 3e). This supports the interpretation that gene *91* is expressed during lytic growth at levels sufficient to kill the host cell before phage replication is complete.

Infection assays showed that the Δ*90* mutant is defective in plaque formation under the standard conditions at 37°C in the absence of gene *90* complementation (Fig. 3f). This phenotype is suppressed when grown at 25°C, with the primary defect being a small reduction in the size of plaques and spots (Fig. 3f); neither WT Adephagia nor any of the mutant derivatives grow well at 42°C (Fig. 3f). We observed that derivatives of the Δ*90* mutant forming plaques on noncomplementing strains at 37°C arise at readily observable frequencies (Fig. 3f, Supplementary Fig. 6a in Supplementary File 1). Sequencing of these Δ*90* derivatives identified several mutants with mutations in gene *91*, including the substitutions R26H, I33T, P42S, P42L, Y52C, K56E, G68D, and R71* (Supplementary Fig. 6a in Supplementary File 1). However, several mutants do not have gene *91* mutations but have base changes immediately upstream of gene *91*, presumably interrupting gene *91* expression (Supplementary Fig. 6a in Supplementary File 1). This is consistent with the interpretation that gp91 toxicity interferes with normal plaque formation of the Δ*90* mutant. The reason for the temperature-dependent phenotype is unclear, and induced expression of *91* from plasmid pDJ14 is toxic at both 25 and 37°C (Supplementary Fig. 6b in Supplementary File 1). Interestingly, we observe that plaque formation of the Δ*90* phage mutant is also influenced by the number of bacterial cells input into the assay (Supplementary Fig. 6c in Supplementary File 1). When 100-fold fewer cells are used, Δ*90* plaques are observed even at 37°C, although they are substantially smaller than at 25°C. It is likely that the number of cycles of bacterial growth on solid media influences the ability of the Δ*90* mutant to form visible plaques.

Both Δ*91* and Δ*90*Δ*91* mutants form lysogens at frequencies similar to that of the parental phage (∼40%) under the assay conditions used (Fig. 3f). The Δ*91* and Δ*90*Δ*91* lysogens grow well, and phage particles are spontaneously released at similar frequencies to the WT phage (Fig. 3g). We also compared the stabilities of the lysogens but observed no differences over the time frames (∼40 generations) we could readily examine (Fig. 3h). We also observed bacterial survivors in this assay with the Δ*90* Adephagia mutant, although with a small colony phenotype and at a frequency reduced at least 10-fold from WT Adephagia. PCR amplification of the gene *91* region of the prophages of these survivors showed that many have mutations in gene *91*, and amino acid substitutions include R26H, H31R, and R4C. These observations suggest that gene *91* is expressed during lysogenic growth, that gene *90* is required for normal establishment of lysogeny, and that lysogens are only recovered from the subpopulation of mutants that suppress the defect resulting from loss of gene *90*.

### Predicted structures of Adephagia gp90 and gp91

AlphaFold2 predictions of Adephagia gp90 and gp91 reveal structures with strong similarities to other HicAB proteins (Fig. 4; Gerdes 2024). The 71-residue Adephagia gp91 protein is predicted to have 3 alpha helices at the N- and C-terminal ends flanking 3 beta strands (Fig. 4a) similar to other HicA-like proteins (Fig. 4, a and b). Similarly, the Adephagia 65-residue gp90 protein is predicted to have 4 beta strands flanking 2 alpha helices, similar to other HicB-like proteins (Fig. 4, a and b). The gp91 toxin is substantially basic in its overall charge, consistent with its binding to a nucleic acid target, whereas the gp90 antitoxin is acidic overall (Fig. 4c). Modeling of the 2 proteins as a complex reveals an intimate interface between gp90 and gp91, with 198 close contacts and 15 hydrogen bonds reinforcing the interaction according to ChimeraX structural analysis (Fig. 4d). This interaction leads to a buried surface of 927.8 Å^2^ according to the PISA webserver (Krissinel and Henrick 2007), about 20% of the total solvent-accessible surface area of each protein. The putative catalytic residue H31 is located within the buried surface (Fig. 4, b and d), suggesting that gp90 antitoxin binding to gp91 prevents toxicity by inhibiting catalytically productive interaction between gp91 and its target. The gp90 and gp91 models are structurally similar to the class 2 HicA and HicB proteins encoded by *Campylobacter* sp. RM12654 (Gerdes 2024), and ChimeraX matchmaker alignments of the cognate proteins yield RMSD values of 1.229 for the HicA-like toxins (between 19 pruned atom pairs; 4.431 across all 58 pairs) and 1.040 for the HicB-like antitoxins (between 37 pruned pairs; 4.958 across all 47 aligned pairs; Fig. 4e). Seven single-amino acid substitutions that render gp91 nontoxic, recovered by plating the Adephagia Δ*90* mutant phage on a noncomplementing strain at 37°C, are mapped onto the predicted structure (Fig. 4f). Three additional single-amino acid substitutions that render gp91 nontoxic, recovered by establishing lysogens of the Δ*90* mutant with point mutations to gene *91*, are similarly mapped (Fig. 4g).

## Discussion

We have described here several aspects of *Mycobacterium* phage Adephagia that advance our understanding of its life cycles, gene expression profiles, and gene functions. Adephagia infects many strains of *M. tuberculosis* and some strains of *M. abscessus* (Guerrero-Bustamante *et al*. 2021), and lytic derivatives in which the repressor and integrase genes have been deleted may be of therapeutic utility (Guerrero-Bustamante *et al*. 2021; Hatfull 2023). Elucidation of the gene expression and gene function profiles will advance its potential for therapeutic use.

The Adephagia gene expression profile in lytic growth is similar to that described for other cluster K phages such as ZoeJ (Dedrick, Guerrero Bustamante, Garlena, Pinches *et al*. 2019b), although there are some notable differences. In both ZoeJ and Adephagia, there are variable expression levels of the early lytic genes, with late gene expression beginning near—and to the left—of the right cos-end of the genome (Fig. 1b). In lysogeny, the ZoeJ repressor is expressed at a higher level than it is in Adephagia and a level similar to integrase expression (Fig. 1b; Dedrick, Guerrero Bustamante, Garlena, Pinches *et al*. 2019b). ZoeJ codes for homologues of Adephagia gp36, gp37, and gp38, (also, gp36, gp37, and gp38), and in both genomes, these genes are expressed lysogenically. ZoeJ does not code for a TA system, but a set of 8–10 genes near the right end of the ZoeJ genome are expressed lysogenically (Dedrick, Guerrero Bustamante, Garlena, Pinches *et al*. 2019b). Adephagia has homologues of 4 of these genes, although lysogenic expression was not detected.

Twenty-five of the Adephagia genes tested were shown to be toxic or inhibitory to *M. smegmatis* growth, with the most toxic being genes *35*, *51*, *57*, and *91* (Fig. 1a, Table 1). Genes *35* and *57* code for a putative sliding clamp for DNA replication and a DnaQ-like exonuclease and are likely used for phage DNA replication; their toxicity may derive from interference with the host replication machinery. Gene *51* codes for a 313-residue protein of unknown function, although it contains a DUF3310 domain present in some other phage-encoded proteins; however, homologues are only present in cluster K among the *M. smegmatis* phages. Of interest is the SaV protein of lactococcal phages that also has this domain and interacts with a host abortive infection protein (AbiV) to influence phage protein translation (Haaber *et al*. 2010). It is tempting to speculate that Adephagia gp51 could be involved with translation of those genes carrying an SAS sequence. Another gene of interest is *58* coding for an 88-residue protein of unknown function. There are homologues of gp58 in other *Mycobacterium* phages, as well as phages that infect *Streptomyces*, *Rhodococcus*, and *Gordonia*. It is a candidate for mediating toxicity through interaction and inactivation of a host protein, although a target has yet to be identified. Other Adephagia proteins in this category are gp48, gp54, gp71, and gp81.

TA systems are implicated in phage defense (LeRoux and Laub 2022), and genomes within many different clusters of actinobacteriophages are predicted to encode TA systems (Dedrick *et al*. 2017; Russell and Hatfull 2017). Some are encoded close to the immunity and integration genes and have been shown to be lysogenically expressed (Dedrick *et al*. 2017). In Adephagia, expression of the gp91 toxin is very toxic to *M. smegmatis*, and a Δ*90* mutant is defective in lytic growth and plaque formation under standard plating conditions at 37°C. Similarly, the Δ*90* mutant does not readily integrate into the host to form a lysogen unless there are compensating mutations. This growth impairment in both lytic and lysogenic phases is likely due to gp91 toxicity and can be alleviated by mutations that inactivate gp91 or inhibit its expression (Supplementary Fig. 6a in Supplementary File 1). The prevalence of these mutations limits characterization of the Δ90 mutant to experiments that do not require large numbers of particles. The observed temperature dependence of the toxicity is unexpected and somewhat surprising. It is not due to cold sensitivity of gp91, because the gp91 toxicity is observed at 25°C in a recombinant strain (Supplementary Fig. 6b in Supplementary File 1). Presumably, either the expression of gene *91* from infecting phage particles is impaired at lower temperature, the gp91 target is not available or not required at 25°C, or, as our preliminary data suggest, the phenotype is influenced by bacterial growth rate (Supplementary Fig. 6c in Supplementary File 1). Adephagia has a relatively restricted growth range and does not form plaques at 42°C (Fig. 3f, Supplementary Fig. 6b in Supplementary File 1), and other cluster K phages have been reported to only grow at lower temperatures (Pope *et al*. 2011). *Mycobacterium* strains vary greatly in their range of preferred growth temperatures, and it is plausible that cluster K phages have evolved in specific host strains and environments at temperatures in the 25–30°C span.

Deletion of the TA system does not appear to compromise prophage stability, and we favor the interpretation that it confers defense against superinfecting heterotypic phages, although we could not identify any targeting phages among a diverse panel of phages that we tested. However, defense could be very specific, and phages that trigger abortive infection through inactivation of the gp90 antitoxin have yet to be identified or tested.

The Adephagia TA system could be a useful tool for advancing genetic systems for *Mycobacterium* strains. It is self-contained within a small 566 bp region containing the two open reading frames, the intergenic regulator sequences, and the putative P_90_ promoter. Extrachromosomal plasmids based on the pAL5000 replicon are commonly used in *Mycobacterium* genetics but are often unstable and lost during unselected growth (Lee *et al*. 1991). Addition of the Adephagia TA system may promote inherent plasmid stability even without antibiotic selection, an advantage for in vivo experiments for understanding pathogenicity.

## Supplementary Material

jkae166_Supplementary_Data

## Data Availability

All materials including plasmids, phages, and protein models are available upon request. RNA-seq are deposited and publicly available at the Gene Expression Omnibus (accession number GSE271014). Supplemental material available at G3 online.
