# Supplementary material for: Characterization of mycobacteriophage Adephagia cytotoxic proteins
Source: G3 (Bethesda). 2024 Jul 20;14(9):jkae166. doi: 10.1093/g3journal/jkae166 (PMC11373665; doi:10.1093/g3journal/jkae166)
Supplement: jkae166_Supplementary_Data [file jkae166_supplementary_data.pdf]

Supplementary Materials

Supplementary Tables

Table S1. Plasmids reported in this study.

Supplemental Figure legends

Figure S1. Adephagia protein expression and solubility assay. Mycobacteriophage Adephagia proteins were cloned as N-terminal fusions to mRuby2 and overexpressed in *E. coli*. Cultures were lysed to create a whole cell lysate (W) and centrifuged to isolate a soluble fraction (S) in the supernatant. The solubility of each protein after autoinduction at 17 °C (gray) or 37 °C (orange) was assessed with plate reader assays quantifying the mRuby2 signal in the soluble fraction versus the whole cell lysate, **a**. The expression and solubility of each protein after expression at 17 °C was assessed by SDS-PAGE analysis, **b**. A marker (M) indicates size standards and the W and S samples of each culture are indicated above each well; the expected size of the fusion protein is shown below each well set.

Figure S2. AlphaFold2 models of Adephagia proteins resulting in cytotoxic and small colony phenotypes in *M. smegmatis* mc^{2}155. a. AlphaFold2-predicted tertiary structures for Adephagia-encoded proteins that are cytotoxic when systematically overexpressed on a Tet-on inducible vector, colored rainbow from N- (blue) to C-terminus (red), and **b.** predicted Adephagia protein models conferring a small colony phenotype to ATc-induced colonies. The size of each model is scaled relative to each structure.

Figure S3. Raw data for cytotoxicity biological triplicates. Biological triplicates of the assay shown in Figure 2a, demonstrating log-reductions in colony growth on uninduced and induced

plates as indicated at the bottom of the figure. The protein being expressed (or the empty vector control) is indicated above each set of plates. Next to each biological replicate are cytotoxicity scores of '++', '+++', '++++', or '+++++', indicating 2-5 log reduction in viability, respectively.

Figure S4. Small colony phenotype analysis. *M. smegmatis* mc^2155 transformants carrying expression vectors encoding Adephagia genes that demonstrate small colony phenotypes were diluted and plated onto solid media with and without 100 ng/ml ATc inducer as indicated. Adephagia proteins are labeled above the corresponding set of plates, along with an empty vector control. Violin plots quantifying the total surface area of each colony-forming unit (cfu) in millimeters-squared on the uninduced (grey) and induced (red) solid medium plates are shown next to each plate set. Significance as determined by an unpaired t-test is denoted with asterisks as follows: $p \leq 0.01$ (**), ≤ 0.001 (***), ≤ 0.0001 (****), ns = not significant.

Figure S5. Adephagia TA-system superinfection defense screen. Mycobacteriophages from 61 different clusters/subclusters, with phage names indicated on the left and cluster/subcluster designation in parentheses, were serially diluted and spotted onto bacterial lawns to assess for superinfection defense. The bacterial strains tested included *M. smegmatis* mc^2155 as a control and lysogens mc^2155(wild-type Adephagia), mc^2155(AdephagiaΔ*91*) and mc^2155(AdephagiaΔ90Δ*91*).

Figure S6. Temperature dependence of TA system. a. The generation of derivatives of AdephagiaΔ*90* which plaque on a non-complementing strain is described. Five independent lysates of AdephagiaΔ*90* (top line) were plated on the complementing strain for single plaques (middle line); two plaques from each initial lysate were propagated as lysates on the complementing strain. The 10 lysates were then plated on a 10X concentrated non-

complementing strain for single plaques and two isolated plaques per lysate were picked (bottom line). The *90-91* regions of these plaque picks were amplified by PCR and sequenced with Sanger sequencing; the character of the resulting gp9 is displayed below each plaque pick and color coded (red for wild type gene *91* sequence, blue for a sequence with missense mutations to gene *91*). These plaques were propagated as lysates on a complementing strain and DNA was extracted and subjected to complete sequencing via NextSeq; the mutations uncovered are indicated beneath the Sanger results and include SNPs and indels in the ESAS region of derivatives with wild type gene *91* sequences. The mutations to gene 91 and the ESAS region (bottom) are summarized in gp91 and ESAS sequences at the bottom of this panel. **b.** Ten-fold serial dilutions of *M. smegmatis* cultures carrying plasmids as shown on the left were plated on solid media either with or without ATc inducer and incubated for three days at 37º C or for nine days at 25º C. **c.** Cells from a 10 ml sample of cultured *M. smegmatis* mc^2155 were pelleted by centrifugation and the supernatant was removed and transferred to a sterile tube. Sufficient volume of the conditioned medium supernatant was added back to the tube and the pellet was resuspended in a total volume of 1 ml. The conditioned medium supernatant was also used to dilute the cell culture by a factor of ten by mixing 900 μl of supernatant with 100 μl of culture. These samples, as well as the neat culture, were infected with 10 μl of the 10^{-5} dilution of an AdephagiaΔ*90* lysate and then mixed with top agar and spread onto 7H10 agar plates to form phage-infected bacterial lawns with different cell densities (10X, 1X, and 0.1X, as indicated above the plates). The plates were incubated overnight at 42° C and 37º C or for five days at 25º C.

Table S1. Plasmids reported in this study

Plasmid[1]	Gene[2]	Abx[R,3]	Replication[4]	Promoter	Vector[5]
pKF8	Vector	Hygromycin B	oriE/oriM	tet-ON (inducible)	pCCK11
pKF114	*mCherry* (expression control)	Hygromycin B	oriE/oriM	tet-ON (inducible)	pKF8
pKF115	*Fruitloop_52* (toxic control)	Hygromycin B	oriE/oriM	tet-ON (inducible)	pKF8
pML95	*Adephagia_1*	Hygromycin B	oriE/oriM	tet-ON (inducible)	pKF8
pML96	*Adephagia_2*	Hygromycin B	oriE/oriM	tet-ON (inducible)	pKF8
pML97	*Adephagia_3*	Hygromycin B	oriE/oriM	tet-ON (inducible)	pKF8
pML98	*Adephagia_4*	Hygromycin B	oriE/oriM	tet-ON (inducible)	pKF8
pML99	*Adephagia_33*	Hygromycin B	oriE/oriM	tet-ON (inducible)	pKF8
pKF165	*Adephagia_34*	Hygromycin B	oriE/oriM	tet-ON (inducible)	pKF8
pKF166	*Adephagia_35*	Hygromycin B	oriE/oriM	tet-ON (inducible)	pKF8
pKF167	*Adephagia_36*	Hygromycin B	oriE/oriM	tet-ON (inducible)	pKF8
pKF168	*Adephagia_37*	Hygromycin B	oriE/oriM	tet-ON (inducible)	pKF8
pKF169	*Adephagia_38*	Hygromycin B	oriE/oriM	tet-ON (inducible)	pKF8
pKF170	*Adephagia_39*	Hygromycin B	oriE/oriM	tet-ON (inducible)	pKF8
pML100	*Adephagia_40*	Hygromycin B	oriE/oriM	tet-ON (inducible)	pKF8
pKF214	*Adephagia_42*	Hygromycin B	oriE/oriM	tet-ON (inducible)	pKF8
pKF171	*Adephagia_43*	Hygromycin B	oriE/oriM	tet-ON (inducible)	pKF8
pKF172	*Adephagia_44*	Hygromycin B	oriE/oriM	tet-ON (inducible)	pKF8
pKF173	*Adephagia_45*	Hygromycin B	oriE/oriM	tet-ON (inducible)	pKF8
pKF174	*Adephagia_46*	Hygromycin B	oriE/oriM	tet-ON (inducible)	pKF8
pML101	*Adephagia_47*	Hygromycin B	oriE/oriM	tet-ON (inducible)	pKF8
pKF175	*Adephagia_48*	Hygromycin B	oriE/oriM	tet-ON (inducible)	pKF8
pKF176	*Adephagia_49*	Hygromycin B	oriE/oriM	tet-ON (inducible)	pKF8
pKF177	*Adephagia_50*	Hygromycin B	oriE/oriM	tet-ON (inducible)	pKF8
pKF178	*Adephagia_51*	Hygromycin B	oriE/oriM	tet-ON (inducible)	pKF8
pDJ6	*Adephagia_52*	Hygromycin B	oriE/oriM	tet-ON (inducible)	pKF8
pDJ7	*Adephagia_53*	Hygromycin B	oriE/oriM	tet-ON (inducible)	pKF8
pKF179	*Adephagia_54*	Hygromycin B	oriE/oriM	tet-ON (inducible)	pKF8
pKF180	*Adephagia_55*	Hygromycin B	oriE/oriM	tet-ON (inducible)	pKF8
pKF181	*Adephagia_56*	Hygromycin B	oriE/oriM	tet-ON (inducible)	pKF8
pKF182	*Adephagia_57*	Hygromycin B	oriE/oriM	tet-ON (inducible)	pKF8
pKF183	*Adephagia_58*	Hygromycin B	oriE/oriM	tet-ON (inducible)	pKF8
pKF268	*Adephagia_59*	Hygromycin B	oriE/oriM	tet-ON (inducible)	pKF8
pDJ8	*Adephagia_60*	Hygromycin B	oriE/oriM	tet-ON (inducible)	pKF8
pKF184	*Adephagia_61*	Hygromycin B	oriE/oriM	tet-ON (inducible)	pKF8
pKF185	*Adephagia_62*	Hygromycin B	oriE/oriM	tet-ON (inducible)	pKF8
pKF186	*Adephagia_63*	Hygromycin B	oriE/oriM	tet-ON (inducible)	pKF8
pKF187	*Adephagia_64*	Hygromycin B	oriE/oriM	tet-ON (inducible)	pKF8
pKF188	*Adephagia_65*	Hygromycin B	oriE/oriM	tet-ON (inducible)	pKF8
pKF189	*Adephagia_66*	Hygromycin B	oriE/oriM	tet-ON (inducible)	pKF8
pDJ9	*Adephagia_67*	Hygromycin B	oriE/oriM	tet-ON (inducible)	pKF8
pKF266	*Adephagia_68*	Hygromycin B	oriE/oriM	tet-ON (inducible)	pKF8
pKF267	*Adephagia_69*	Hygromycin B	oriE/oriM	tet-ON (inducible)	pKF8
pKF190	*Adephagia_70*	Hygromycin B	oriE/oriM	tet-ON (inducible)	pKF8
pKF191	*Adephagia_71*	Hygromycin B	oriE/oriM	tet-ON (inducible)	pKF8
pKF192	*Adephagia_72*	Hygromycin B	oriE/oriM	tet-ON (inducible)	pKF8
pKF193	*Adephagia_73*	Hygromycin B	oriE/oriM	tet-ON (inducible)	pKF8
pKF194	*Adephagia_74*	Hygromycin B	oriE/oriM	tet-ON (inducible)	pKF8
pKF195	*Adephagia_75*	Hygromycin B	oriE/oriM	tet-ON (inducible)	pKF8
pDJ10	*Adephagia_76*	Hygromycin B	oriE/oriM	tet-ON (inducible)	pKF8
pKF196	*Adephagia_77*	Hygromycin B	oriE/oriM	tet-ON (inducible)	pKF8
pDJ11	*Adephagia_78*	Hygromycin B	oriE/oriM	tet-ON (inducible)	pKF8
pKF197	*Adephagia_79*	Hygromycin B	oriE/oriM	tet-ON (inducible)	pKF8
pKF198	*Adephagia_80*	Hygromycin B	oriE/oriM	tet-ON (inducible)	pKF8
pKF199	*Adephagia_81*	Hygromycin B	oriE/oriM	tet-ON (inducible)	pKF8
pKF200	*Adephagia_82*	Hygromycin B	oriE/oriM	tet-ON (inducible)	pKF8
pKF201	*Adephagia_83*	Hygromycin B	oriE/oriM	tet-ON (inducible)	pKF8
pDJ12	*Adephagia_84*	Hygromycin B	oriE/oriM	tet-ON (inducible)	pKF8
pKF203	*Adephagia_85*	Hygromycin B	oriE/oriM	tet-ON (inducible)	pKF8
pKF204	*Adephagia_86*	Hygromycin B	oriE/oriM	tet-ON (inducible)	pKF8

pKF205	*Adephagia_87*	Hygromycin B	oriE/oriM	tet-ON (inducible)	pKF8
pKF206	*Adephagia_88*	Hygromycin B	oriE/oriM	tet-ON (inducible)	pKF8
pKF207	*Adephagia_89*	Hygromycin B	oriE/oriM	tet-ON (inducible)	pKF8
pKF208	*Adephagia_90*	Hygromycin B	oriE/oriM	tet-ON (inducible)	pKF8
pKF209	*Adephagia_91*	Hygromycin B	oriE/oriM	tet-ON (inducible)	pKF8
pKF210	*Adephagia_92*	Hygromycin B	oriE/oriM	tet-ON (inducible)	pKF8
pDJ13	*Adephagia_93*	Hygromycin B	oriE/oriM	tet-ON (inducible)	pKF8
pKF212	*Adephagia_94*	Hygromycin B	oriE/oriM	tet-ON (inducible)	pKF8
pKF213	*Adephagia_95*	Hygromycin B	oriE/oriM	tet-ON (inducible)	pKF8
pKF7	Vector	Streptomycin	oriE/attP-int	tet-ON (inducible)	pCCK41
pDJ14	*Adephagia_91*	Streptomycin	oriE/attP-int	tet-ON (inducible)	pKF7
pDJ15	*Adephagia_91 G28C mutant*	Streptomycin	oriE/attP-int	tet-ON (inducible)	pKF7
pDJ16	*Adephagia_91 H31A mutant*	Streptomycin	oriE/attP-int	tet-ON (inducible)	pKF7
pDJ17	*Adephagia_90 T31V mutant*	Hygromycin B	oriE/oriM	tet-ON (inducible)	pKF8
pDJ18	*Adephagia_90*	Kanamycin	oriE/attP-int	HSP-60 (constitutive)	pLO74
pKF116	*Adephagia_34*	Kanamycin	oriE	T7 (inducible)	pET28a
pKF117	*Adephagia_35*	Kanamycin	oriE	T7 (inducible)	pET28a
pKF118	*Adephagia_36*	Kanamycin	oriE	T7 (inducible)	pET28a
pKF119	*Adephagia_37*	Kanamycin	oriE	T7 (inducible)	pET28a
pKF120	*Adephagia_38*	Kanamycin	oriE	T7 (inducible)	pET28a
pKF121	*Adephagia_39*	Kanamycin	oriE	T7 (inducible)	pET28a
pKF122	*Adephagia_43*	Kanamycin	oriE	T7 (inducible)	pET28a
pKF123	*Adephagia_44*	Kanamycin	oriE	T7 (inducible)	pET28a
pKF124	*Adephagia_45*	Kanamycin	oriE	T7 (inducible)	pET28a
pKF125	*Adephagia_46*	Kanamycin	oriE	T7 (inducible)	pET28a
pKF126	*Adephagia_48*	Kanamycin	oriE	T7 (inducible)	pET28a
pKF127	*Adephagia_49*	Kanamycin	oriE	T7 (inducible)	pET28a
pKF128	*Adephagia_50*	Kanamycin	oriE	T7 (inducible)	pET28a
pKF129	*Adephagia_51*	Kanamycin	oriE	T7 (inducible)	pET28a
pKF130	*Adephagia_54*	Kanamycin	oriE	T7 (inducible)	pET28a
pKF131	*Adephagia_55*	Kanamycin	oriE	T7 (inducible)	pET28a
pKF132	*Adephagia_56*	Kanamycin	oriE	T7 (inducible)	pET28a
pKF133	*Adephagia_57*	Kanamycin	oriE	T7 (inducible)	pET28a
pKF134	*Adephagia_58*	Kanamycin	oriE	T7 (inducible)	pET28a
pKF135	*Adephagia_61*	Kanamycin	oriE	T7 (inducible)	pET28a
pKF136	*Adephagia_62*	Kanamycin	oriE	T7 (inducible)	pET28a
pKF137	*Adephagia_63*	Kanamycin	oriE	T7 (inducible)	pET28a
pKF138	*Adephagia_64*	Kanamycin	oriE	T7 (inducible)	pET28a
pKF139	*Adephagia_65*	Kanamycin	oriE	T7 (inducible)	pET28a
pKF140	*Adephagia_66*	Kanamycin	oriE	T7 (inducible)	pET28a
pKF141	*Adephagia_70*	Kanamycin	oriE	T7 (inducible)	pET28a
pKF142	*Adephagia_71*	Kanamycin	oriE	T7 (inducible)	pET28a
pKF143	*Adephagia_72*	Kanamycin	oriE	T7 (inducible)	pET28a
pKF144	*Adephagia_73*	Kanamycin	oriE	T7 (inducible)	pET28a
pKF145	*Adephagia_74*	Kanamycin	oriE	T7 (inducible)	pET28a
pKF146	*Adephagia_75*	Kanamycin	oriE	T7 (inducible)	pET28a
pKF147	*Adephagia_77*	Kanamycin	oriE	T7 (inducible)	pET28a
pKF148	*Adephagia_79*	Kanamycin	oriE	T7 (inducible)	pET28a
pKF149	*Adephagia_80*	Kanamycin	oriE	T7 (inducible)	pET28a
pKF150	*Adephagia_81*	Kanamycin	oriE	T7 (inducible)	pET28a
pKF151	*Adephagia_82*	Kanamycin	oriE	T7 (inducible)	pET28a
pKF152	*Adephagia_83*	Kanamycin	oriE	T7 (inducible)	pET28a
pKF153	*Adephagia_84*	Kanamycin	oriE	T7 (inducible)	pET28a
pKF154	*Adephagia_85*	Kanamycin	oriE	T7 (inducible)	pET28a
pKF155	*Adephagia_86*	Kanamycin	oriE	T7 (inducible)	pET28a
pKF156	*Adephagia_87*	Kanamycin	oriE	T7 (inducible)	pET28a
pKF157	*Adephagia_88*	Kanamycin	oriE	T7 (inducible)	pET28a
pKF158	*Adephagia_89*	Kanamycin	oriE	T7 (inducible)	pET28a
pKF159	*Adephagia_90*	Kanamycin	oriE	T7 (inducible)	pET28a
pKF160	*Adephagia_91*	Kanamycin	oriE	T7 (inducible)	pET28a
pKF161	*Adephagia_92*	Kanamycin	oriE	T7 (inducible)	pET28a
pKF162	*Adephagia_93*	Kanamycin	oriE	T7 (inducible)	pET28a

pKF163	*Adephagia_94*	Kanamycin	oriE	T7 (inducible)	pET28a
pKF164	*Adephagia_95*	Kanamycin	oriE	T7 (inducible)	pET28a

[1]Plasmid name
[2]Gene inserted into vector
[3]Antibiotic resistance cassette in vector
[4]Replication system, oriE, oriM or attP-Int derived from phage L5
[5]Plasmid vector
*Sequences for the *Adephagia_90* protospacer were produced by the Mycobacterial CRISPRi Primer Design program, and the two annealed oligo sequences used to assemble pDJ5 are as follows: 5'-GGGAGCAGTGGACGCAAGCCCGTAG-3' and 5'-AAACCTACGGGCTTGCGTCCACTGC-3'.

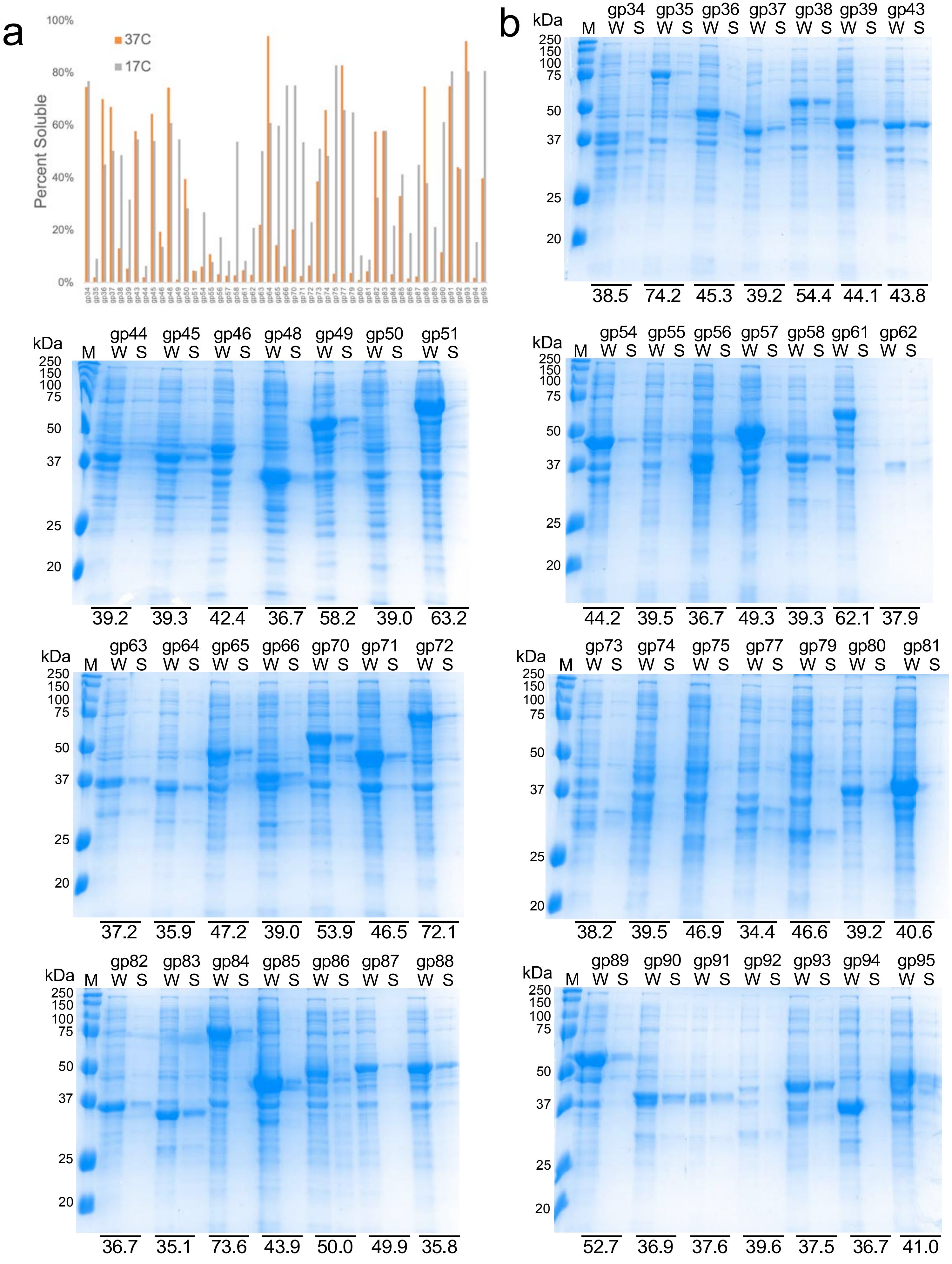
a
37C
17C
Percent Soluble
100%
80%
60%
40%
20%
0%
b
kDa
M
250
150
100
75
50
37
25
20
gp34 W S
gp35 W S
gp36 W S
gp37 W S
gp38 W S
gp39 W S
gp43 W S
38.5 74.2 45.3 39.2 54.4 44.1 43.8
gp44 W S
gp45 W S
gp46 W S
gp48 W S
gp49 W S
gp50 W S
gp51 W S
39.2 39.3 42.4 36.7 58.2 39.0 63.2
gp54 W S
gp55 W S
gp56 W S
gp57 W S
gp58 W S
gp61 W S
gp62 W S
44.2 39.5 36.7 49.3 39.3 62.1 37.9
gp63 W S
gp64 W S
gp65 W S
gp66 W S
gp70 W S
gp71 W S
gp72 W S
37.2 35.9 47.2 39.0 53.9 46.5 72.1
gp73 W S
gp74 W S
gp75 W S
gp77 W S
gp79 W S
gp80 W S
gp81 W S
38.2 39.5 46.9 34.4 46.6 39.2 40.6
gp82 W S
gp83 W S
gp84 W S
gp85 W S
gp86 W S
gp87 W S
gp88 W S
36.7 35.1 73.6 43.9 50.0 49.9 35.8
gp89 W S
gp90 W S
gp91 W S
gp92 W S
gp93 W S
gp94 W S
gp95 W S
52.7 36.9 37.6 39.6 37.5 36.7 41.0

Figure S1

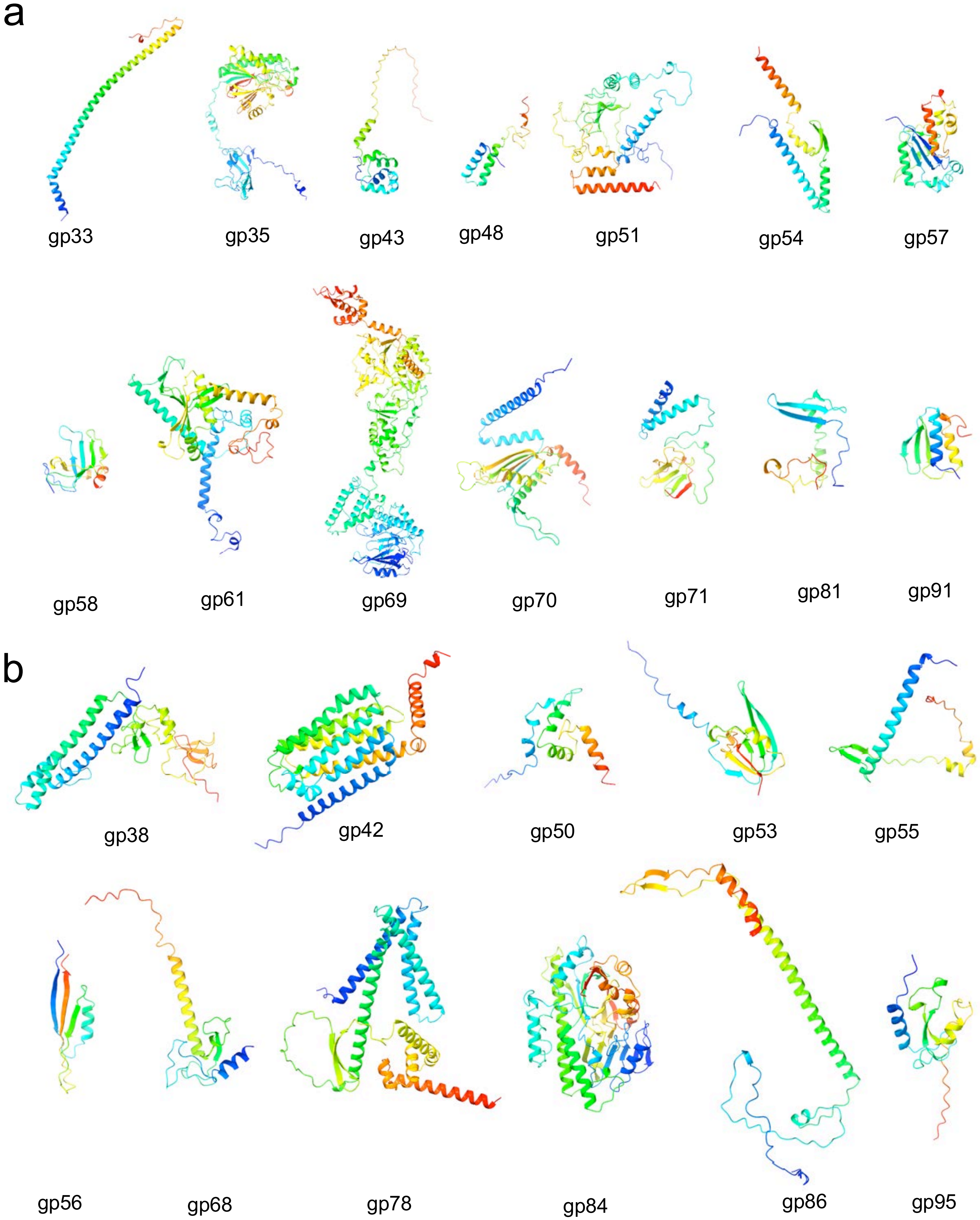
a
gp33
gp35
gp43
gp48
gp51
gp54
gp57
gp58
gp61
gp69
gp70
gp71
gp81
gp91
b
gp38
gp42
gp50
gp53
gp55
gp56
gp68
gp78
gp84
gp86
gp95

Figure S2

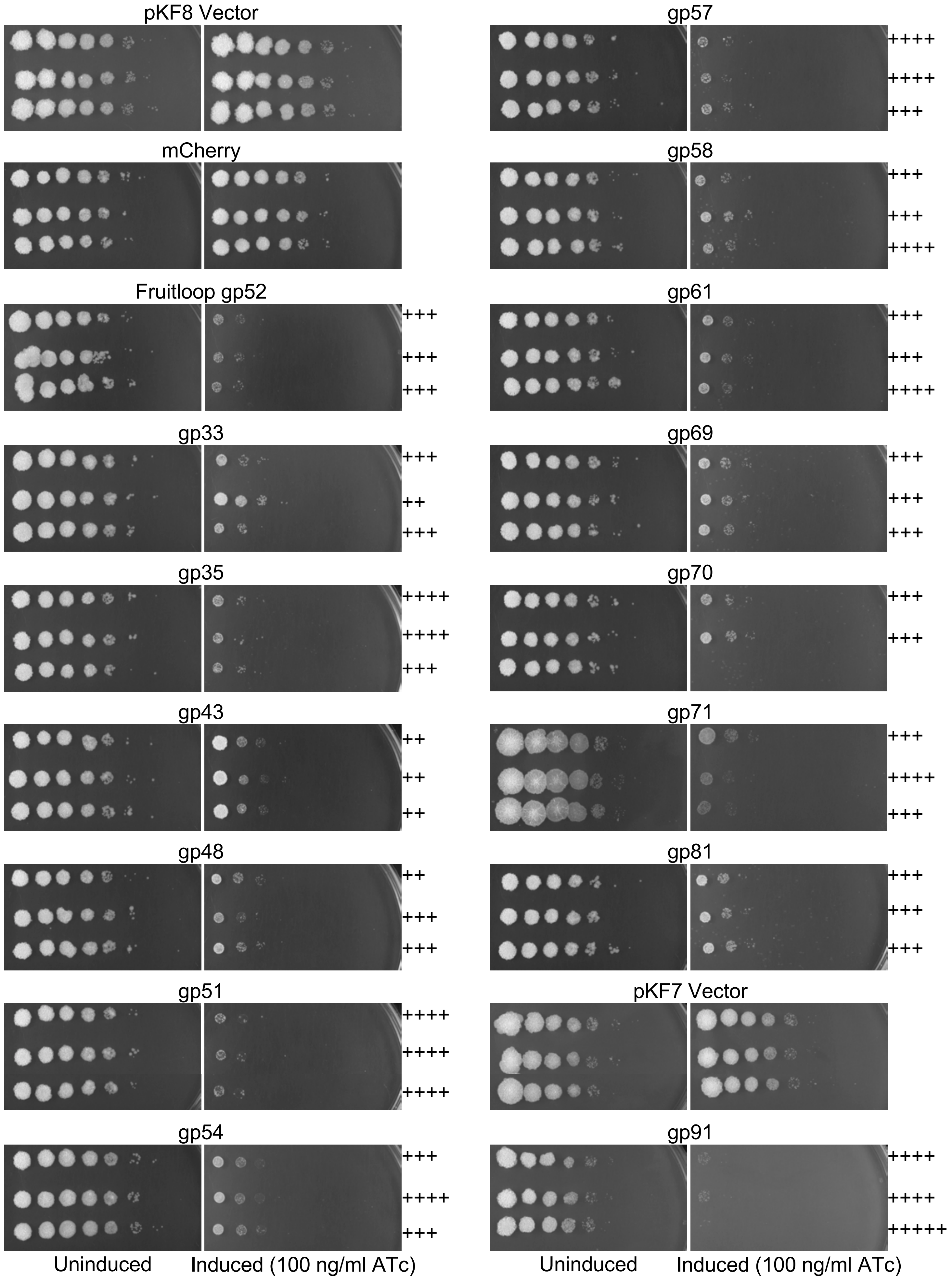
pKF8 Vector
mCherry
Fruitloop gp52
+++
+++
+++
gp33
+++
++
+++
gp35
++++
++++
+++
gp43
++
++
++
gp48
++
+++
+++
gp51
++++
++++
++++
gp54
+++
++++
+++
gp57
++++
++++
+++
gp58
+++
+++
++++
gp61
+++
+++
++++
gp69
+++
+++
+++
gp70
+++
+++
gp71
+++
++++
+++
gp81
+++
+++
+++
pKF7 Vector
gp91
++++
++++
+++++
Uninduced
Induced (100 ng/ml ATc)
Uninduced
Induced (100 ng/ml ATc)

Figure S3

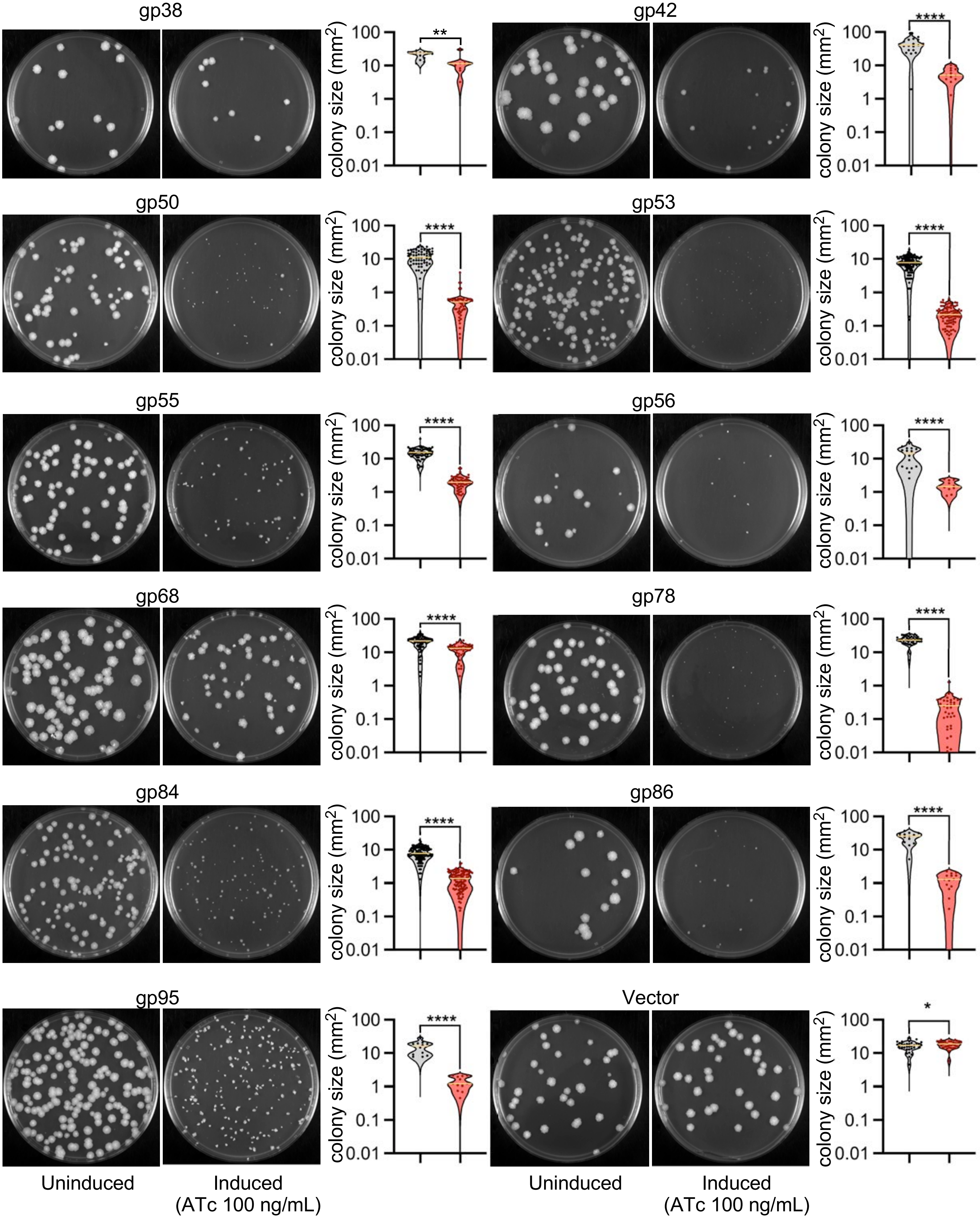
gp38
gp42
gp50
gp53
gp55
gp56
gp68
gp78
gp84
gp86
gp95
Vector
colony size (mm^2)
100
10
1
0.1
0.01
**
****
*
Uninduced
Induced
(ATc 100 ng/mL)

Figure S4

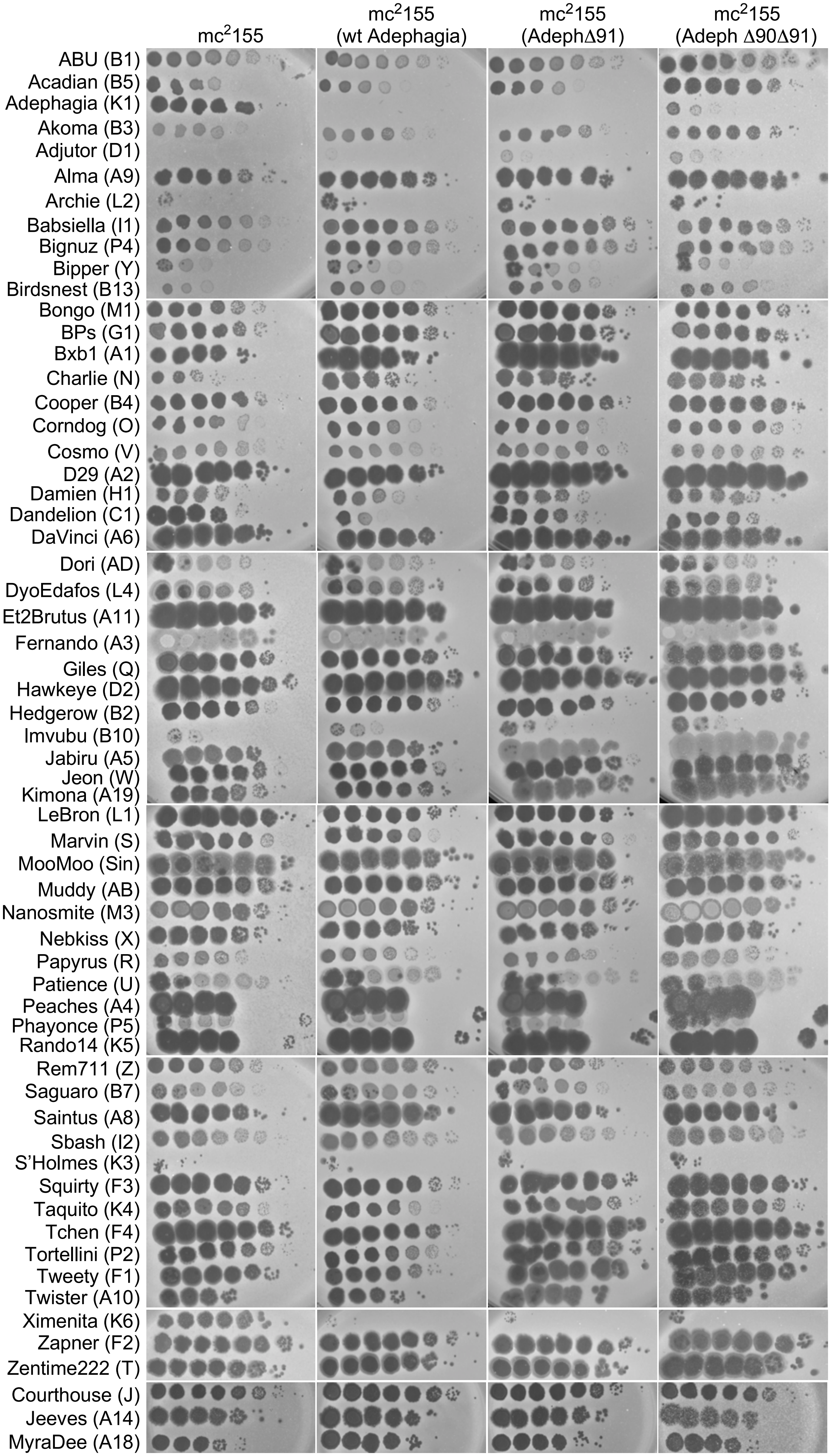
mc²155
mc²155 (wt Adephagia)
mc²155 (AdephΔ91)
mc²155 (Adeph Δ90Δ91)
ABU (B1)
Acadian (B5)
Adephagia (K1)
Akoma (B3)
Adjutor (D1)
Alma (A9)
Archie (L2)
Babsiella (I1)
Bignuz (P4)
Bipper (Y)
Birdsnest (B13)
Bongo (M1)
BPs (G1)
Bxb1 (A1)
Charlie (N)
Cooper (B4)
Corndog (O)
Cosmo (V)
D29 (A2)
Damien (H1)
Dandelion (C1)
DaVinci (A6)
Dori (AD)
DyoEdafos (L4)
Et2Brutus (A11)
Fernando (A3)
Giles (Q)
Hawkeye (D2)
Hedgerow (B2)
Imvubu (B10)
Jabiru (A5)
Jeon (W)
Kimona (A19)
LeBron (L1)
Marvin (S)
MooMoo (Sin)
Muddy (AB)
Nanosmite (M3)
Nebkiss (X)
Papyrus (R)
Patience (U)
Peaches (A4)
Phayonce (P5)
Rando14 (K5)
Rem711 (Z)
Saguaro (B7)
Saintus (A8)
Sbash (I2)
S'Holmes (K3)
Squirty (F3)
Taquito (K4)
Tchen (F4)
Tortellini (P2)
Tweety (F1)
Twister (A10)
Ximenita (K6)
Zapner (F2)
Zentime222 (T)
Courthouse (J)
Jeeves (A14)
MyraDee (A18)

Figure S5

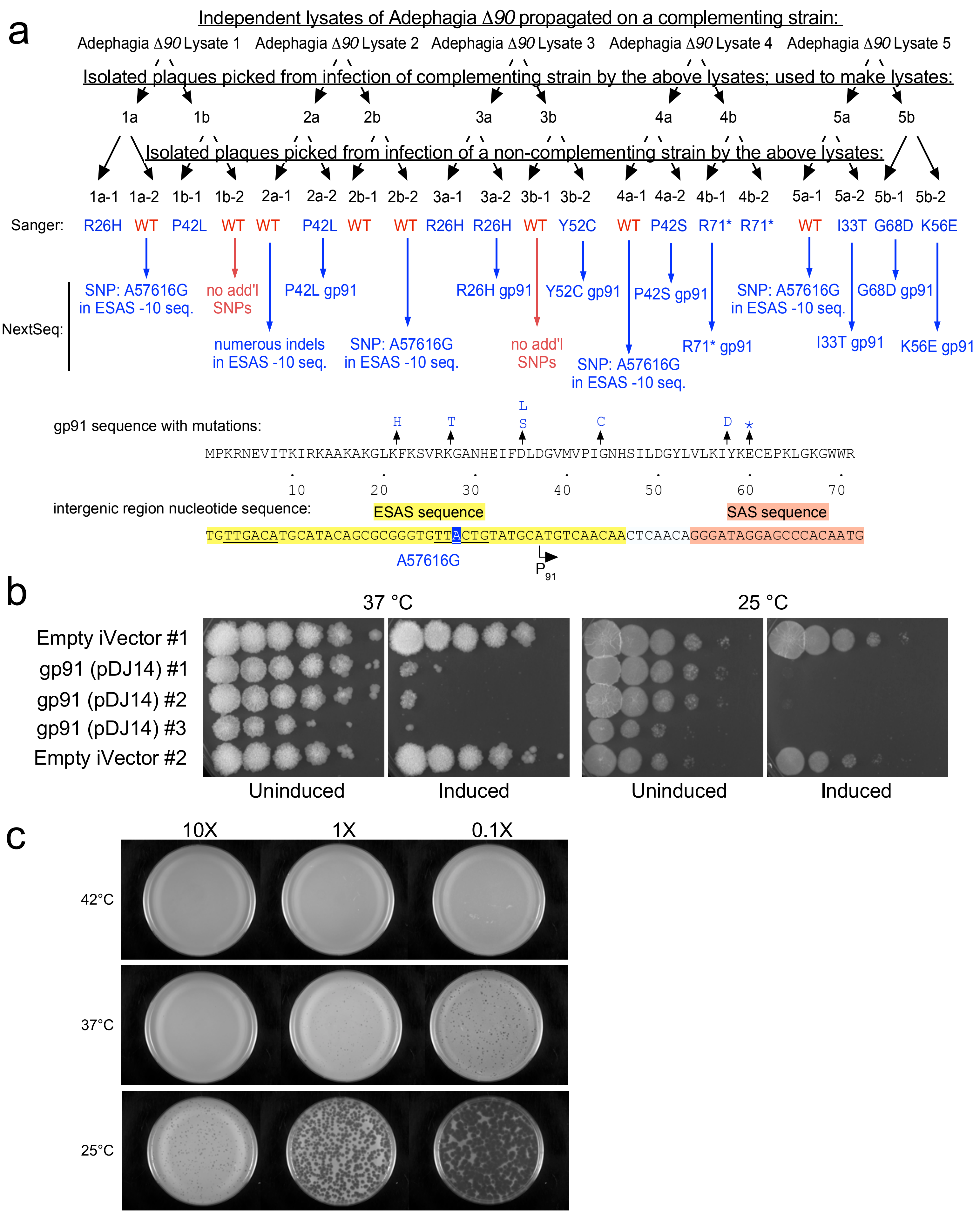
a
Independent lysates of Adephagia Δ90 propagated on a complementing strain:
Adephagia Δ90 Lysate 1
Adephagia Δ90 Lysate 2
Adephagia Δ90 Lysate 3
Adephagia Δ90 Lysate 4
Adephagia Δ90 Lysate 5
Isolated plaques picked from infection of complementing strain by the above lysates; used to make lysates:
1a 1b 2a 2b 3a 3b 4a 4b 5a 5b
Isolated plaques picked from infection of a non-complementing strain by the above lysates:
1a-1 1a-2 1b-1 1b-2 2a-1 2a-2 2b-1 2b-2 3a-1 3a-2 3b-1 3b-2 4a-1 4a-2 4b-1 4b-2 5a-1 5a-2 5b-1 5b-2
Sanger:
R26H WT P42L WT WT P42L WT WT R26H R26H WT Y52C WT P42S R71* R71* WT I33T G68D K56E
NextSeq:
SNP: A57616G in ESAS -10 seq.
no add'l SNPs
P42L gp91
numerous indels in ESAS -10 seq.
SNP: A57616G in ESAS -10 seq.
R26H gp91
no add'l SNPs
Y52C gp91
P42S gp91
SNP: A57616G in ESAS -10 seq.
R71* gp91
SNP: A57616G in ESAS -10 seq.
G68D gp91
I33T gp91
K56E gp91
gp91 sequence with mutations:
H T L S C D *
MPKRNEVITKIRKAAKAKGLKFKSVRKGANHEIFDLDGVMVPIGNHSILDGYLVLKIYKECEPKLGKGWWR
10 20 30 40 50 60 70
intergenic region nucleotide sequence:
ESAS sequence
SAS sequence
TGTTGACATGCATACAGCGCGGGTGTTACTGTATGCATGTCAACAACTCAACAGGGATAGGAGCCCACAATG
A57616G
P91
b
37 °C
25 °C
Empty iVector #1
gp91 (pDJ14) #1
gp91 (pDJ14) #2
gp91 (pDJ14) #3
Empty iVector #2
Uninduced
Induced
Uninduced
Induced
c
10X
1X
0.1X
42°C
37°C
25°C

Figure S6
